# Iterative regularization for constrained minimization formulations of nonlinear inverse problems

**DOI:** 10.1007/s10589-021-00343-x

**Published:** 2021-12-19

**Authors:** Barbara Kaltenbacher, Kha Van Huynh

**Affiliations:** Department of Mathematics, Alpen-Adria-Universität Klagenfurt, Klagenfurt, Austria

**Keywords:** Inverse problems, Iterative regularization, Coefficient identification in elliptic PDEs, Impedance acoustic tomography

## Abstract

In this paper we study the formulation of inverse problems as constrained minimization problems and their iterative solution by gradient or Newton type methods. We carry out a convergence analysis in the sense of regularization methods and discuss applicability to the problem of identifying the spatially varying diffusivity in an elliptic PDE from different sets of observations. Among these is a novel hybrid imaging technology known as impedance acoustic tomography, for which we provide numerical experiments.

## Introduction

Inverse problems usually consist of a model

1 $$\begin{aligned} A(x,u)=0 \end{aligned}$$

where the operator *A* acts on the state *u* of a system and contains unknown parameters *x*, and an observation equation

2 $$\begin{aligned} C(x,u)=y \end{aligned}$$

quantifying the additionally available information that is supposed to allow for identifying the parameters *x*; by a slight notation overload, we will often summarize (*x*, *u*) into a single element, which we again call *x*.

The classical formulation of an inverse problem is as an operator equation

3 $$\begin{aligned} F(x)=y \end{aligned}$$

where usually *x* is the searched for parameter (some coefficient, initial or boundary conditions in a PDE or ODE model) but—in an all-at-once formulation—might as well include the state, i.e., the PDE solution. In a conventional reduced setting $F=C\circ S$ is the concatenation of an observation operator *C* with a parameter-to-state map *S* satisfying $A(x,S(x))=0$, whereas an all-at-once setting considers the inverse problem as a system $$\left\{ \begin{array}{c} A(x,u)=0\\ C(x,u)=y\end{array}\right.$$, which by the above mentioned replacement $x:=(x,u)$ takes the form (3), see, e.g. [14, 15]. All-at-once approaches have been studied for PDE constrained optimization already for many years in, e.g., [25–28, 30, 31] due to their computational advantages: The iterates need not be feasible with respect to the PDE constraint which safes computational effort and potentially allows for larger steps. However, this looseness can also lead to convergence problems and we will actually see this in the most challenging of our numerical test cases, namely the severely ill-posed problem of electric impedance tomography EIT. In this sense we here do not intend to favor any of the formulations but clearly point to their chances and limitations.

We here follow the idea of generalizing this to a formulation of an inverse problem as a constrained minimization problem

4 $$\begin{aligned} \min J(x)\quad \text{ s.t. }\; x\in M, \end{aligned}$$

where in a reduced setting, *x* is the parameter and in an all-at-once-setting $x=(x,u)$ contains both parameter and state. In what follows, it will not be necessary to distinguish between these two cases notationally.

Straightforward instances for equivalent minimization based formulations of (1), (2) are, e.g.,

5 $$\begin{aligned}&\min \tfrac{1}{2} \Vert C(x,u)-y\Vert ^2\quad \text{ s.t. }\;A(x,u)=0,\\&\min \tfrac{1}{2} \Vert A(x,u)\Vert ^2\quad \text{ s.t. }\;C(x,u)=y, \end{aligned}$$

or in the context of (3) comprising both the reduced $F(x)=C(S(x))$ and the all-at-once $$F(x,u)=\left( \begin{array}{c} A(x,u)\\ C(x,u)\end{array}\right)$$ setting simply

6 $$\begin{aligned} \min \tfrac{1}{2} \Vert F(x)-y\Vert ^2. \end{aligned}$$

For further examples of such formulations, see., e.g., [16, 32]. In particular we point to the variational formulation of EIT according to Kohn and Vogelius, see, e.g, [24], which will be extended to further diffusion/impendance identification problems in Sect. 4.

Advantages of such minimization based formulations lie in their great versatility, the straightforward addition of regularization by adding penalty terms (Tikhonov’s method) or imposing constraints (Ivanov’s method), as well as the applicability of efficient optimization methods.

As an alternative to applying standard optimization tools to regularized versions of such minimization based formulations, we here study regularization by iterative methods, that take into account ill-posedness by being stopped early enough to prevent dominance of the propagated noise.

If *J* is differentiable then the first order optimality condition for a minimizer of (4) is

7 $$\begin{aligned} \langle \nabla J(x^\dagger ),x-x^\dagger \rangle \ge 0 \quad \text{ for } \text{ all }\;\, x\in M. \end{aligned}$$

Here *J* is a proper functional acting on a Banach space *X*, and we make the normalization assumption

8 $$\begin{aligned} J\ge 0 \quad \text{ on }\; M \quad \text{ and }\quad J(x^\dagger )=\min _{x\in M}J(x)=0 \end{aligned}$$

for $x^\dagger$ solving the inverse problem, i.e., we assume to know the minimal value of *J* (but of course not the minimizer, which is what we intend to retrieve).

In case of (6), condition (8) means attainability of the exact data, which is in fact a very natural condition in inverse problems, where one assumes the noiseless version of the observations $C(x^\dagger ,u^\dagger )$ to be caused by the true parameter $x^\dagger$ in the model $A(x^\dagger ,u^\dagger )=0$.

Typically inverse problems also in the form (4) are ill-posed in the sense that solutions to (4) do not depend continuously on the data *y* that enters the definition of the cost function *J* and/or of the feasible set *M*. Since in a realistic setting the data is contaminated with measurement noise, i.e., only $y^\delta \approx y$ is given, regularization needs to be employed. We first of all do so by possibly adding some regularizing constraints—in particular we think of bound constraints in the sense of Ivanov regularization—and/or by relaxing constraints like fit to the data in the sense of Morozov regularization. In the context of (5), this, e.g., means that we replace $M=\{x\in X: Cx=y\}$ by $\tilde{M}^\delta =\{x\in X : \Vert Cx-y^\delta \Vert \le \tau \delta \text{ and } \tilde{\mathcal {R}}(x)\le \rho \}$, for the noise level $\delta \ge \Vert y-y^\delta \Vert$, some constants $\tau >1$, $\rho >0$ and some functional $\tilde{\mathcal {R}}$ satisfying $\tilde{\mathcal {R}}(x^\dagger )\le \rho$.

Thus we consider the partly regularized problem

9 $$\begin{aligned} \min J^\delta (x)\quad \text{ s.t. }\; x\in \tilde{M}^\delta \end{aligned}$$

which we intend to solve iteratively, where further regularization is incorporated by early stopping and potentially also by adding regularizing terms during the iteration. As in the above example of $\tilde{M}^\delta$, we will generally assume $x^\dagger$ to be feasible also for this modified problem, and also approximately minimal

10 $$\begin{aligned} &x^\dagger \in \tilde{M}^\delta \text{ and } J^\delta (x^\dagger )\le \eta (\delta ) \quad \text{ for } \text{ all }\;\delta \in (0,\bar{\delta }), \\&\quad \text{ where }\; \eta (\delta )>0\;\text{ and }\;\eta (\delta )\rightarrow 0\;\text{ as } \;\delta \rightarrow 0. \end{aligned}$$

A typical choice of the bound in (10) in case of (6) is $\eta (\delta )\sim \delta ^2$, where $\delta$ is the noise level, cf. Remark 2. With (9) we formally stay in the same setting as in (4) and, like in (8), assume

11 $$\begin{aligned} J^\delta \ge 0\quad \text{ on }\; \tilde{M}^\delta . \end{aligned}$$

The key difference to (4) lies in the fact that $J^\delta$ and $\tilde{M}^\delta$ might depend on the noise level and this will in fact be crucial since we will study convergence as $\delta$ tends to zero.

Since we consider formulations of inverse problems as constrained minimization problems, an essential step is to extend iterative regularization methods such as gradient or Newton type methods, to more general cost functions and constrained minimization problems. Clearly, the optimization literature on Newton and gradient methods is vast, however, their application to ill-posed problems requires special consideration. In particular, second order sufficient conditions will typically fail to hold here. To see this, consider the Hessian of the cost function (6) at a solution $J''(x^\dagger )(h,h)=\Vert F'(x^\dagger )h\Vert ^2$. Assuming its uniform positivity amounts to demanding bounded invertibitily of $F'(x^\dagger )$, which usually does not hold for ill-posed problems. Along with these two paradigms concerning the search direction, we will consider two approaches for guaranteeing feasibility of the sequence, namely projection onto the admissible set in the context of gradient methods in Sect. 2 and sequential quadratic programming SQP type constrained minimization in Sect. 3.

Some key references for gradient, i.e., Landweber type iterative methods are [5] on projected Landweber iteration for linear inverse problems, [9] on (unconstrained) nonlinear Landweber iteration, and more recently [23] on gradient type methods under very general conditions on the cost function or the forward operator, respectively. Extensions with a penalty term (also allowing for the incorporation of constraints) for linear inverse problems can be found in [4]; For nonlinear problems we also point to [13, 33], however, they do not seem to be applicable to constrained problems, since the penalty term is assumed to be *p*-convex and thus cannot be an indicator function.

Newton type methods for the solution of nonlinear ill-posed problems have been extensively studied in Hilbert spaces (see, e.g., [3, 19] and the references therein) and more recently also in a in Banach space setting (see, e.g., [29] and the references therein). In particular, the iteratively regularized Gauss–Newton method [2] or the Levenberg–Marquardt method [8] easily allow to incorporate constraints in their variational form. Projected Gauss–Newton type methods for constrained ill-posed problems have been considered in, e.g., [18].

The remainder of this paper is organized as follows. In Sect. 2 we will study a projected version of Landweber iteration, thus a gradient type method in a Hilbert space setting and prove its convergence under certain convexity assumptions on the cost function. Section 3 turns to a general Banach space setting and discusses Newton SQP methods as well as their convergence. Finally, in Sect. 4 we investigate applicability to the identification of a spatially varying diffusion coefficient in an elliptic PDE from different sets of boundary conditions which leads to three different inverse problems: Inverse groundwater filtration (often also used as a model problem and denoted by *a*-problem), impedance acoustic tomography and electrical impedance tomography. Numerical experiments in Sect. 5 illustrate our theoretical findings.

The message of this paper is supposed to be two fold: First of all, we show that for inverse problems formulated by constrained minimization, besides the approach of regularizing and then applying state-of-the-art iterative optimization tools (regularize, then iterate) there is also the option of applying iterative methods to the un- or only partly regularized problem (regularize *by* iteration; more precisely, by early stopping). Secondly, by means of the mentioned diffusion identification problems we wish to demonstrate the large variety of possible minimization formulations arising even in the context of a single elliptic PDE, and to highlight some of the chances and limitations related to these various formulations.

## A projected gradient method

In this section, we consider the projected gradient method for (9)

12 $$\begin{aligned} \tilde{x}_{k+1}=x_k-\mu _k\nabla J^\delta (x_k), \quad x_{k+1}=P_{\tilde{M}^\delta }(\tilde{x}_{k+1}) \end{aligned}$$

and extend some of the results from [23] to the constrained setting, or from a different viewpoint, extend some of the results from [5] to the nonlinear setting. In (12), $\mu _k>0$ is a stepsize parameter and $\nabla J^\delta (x_k)\in X$ is the Riesz representation of ${J^\delta }'(x_k)\in X^*$ as in this section we restrict ourselves to a Hilbert space setting. The reason for this is the fact that in general Banach spaces, ${J^\delta }'(x_k)$ would have to be transported back into *X* by some (nonlinear, e.g. in $L^p$ with $p\not =2$) duality mapping, which adds nonlinearity and therefore, among others, complicates the choice of the step size, see e.g. [21] for the unconstrained least squares case (6). Moreover, throughout this section we will assume $\tilde{M}^\delta$ to be closed and convex and denote by $P_{\tilde{M}^\delta }$ the metric (in the Hilbert space setting considered in this section also orthogonal) projection onto $\tilde{M}^\delta$, which is characterized by the variational inequality

13 $$\begin{aligned} x=P_{\tilde{M}^\delta }(\tilde{x}) \Leftrightarrow \left( x\in \tilde{M}^\delta \;\text{ and }\; \forall z\in \tilde{M}^\delta : \langle \tilde{x}-x,z-x\rangle \le 0 \right) \end{aligned}$$

With $z:=x_k\in \tilde{M}^\delta$, this immediately implies

$$\begin{aligned} 0\ge \langle \tilde{x}_{k+1}-x_{k+1},x_k-x_{k+1}\rangle = \langle x_k-x_{k+1}-\mu _k \nabla J^\delta (x_k),x_k-x_{k+1}\rangle \end{aligned}$$

hence

14 $$\begin{aligned} \Vert x_{k+1}-x_k\Vert ^2\le -\mu _k \langle \nabla J^\delta (x_k),x_{k+1}-x_k\rangle \end{aligned}$$

and thus, using the Cauchy–Schwarz inequality, the estimate

15 $$\begin{aligned} \Vert x_{k+1}-x_k\Vert \le \mu _k \Vert \nabla J^\delta (x_k)\Vert . \end{aligned}$$

Moreover, as well known for (projected) gradient methods, under the Lipschitz type condition on the gradient

16 $$\begin{aligned} J^\delta (x)-J^\delta (x_+)-\langle \nabla J^\delta (x)(x-x_+)\ge -\tfrac{L}{2}\Vert x-x_+\Vert ^2 \quad \text{ for } \text{ all }\; x,x_+\in \tilde{M}^\delta \end{aligned}$$

for $\mu _k\le \overline{\mu }<\frac{2}{L}$, from (14) we get monotonicity of the cost function values

$$\begin{aligned} J^\delta (x_k)-J^\delta (x_{k+1})\ge \left( \tfrac{1}{\mu _k}-\tfrac{L}{2}\right) \Vert x_{k+1}-x_k\Vert ^2 \end{aligned}$$

and square summability of the steps

$$\begin{aligned} \sum _{k=0}^\infty \Vert x_{k+1}-x_k\Vert ^2 \le \frac{1}{\tfrac{1}{\overline{\mu }}-\tfrac{L}{2}} J^\delta (x_0). \end{aligned}$$

Monotonicity of the error under additional convexity assumptions easily follows from nonexpansivity of the projection, which yields

17 $$\begin{aligned} &\Vert x_{k+1}-x^\dagger \Vert ^2-\Vert x_k-x^\dagger \Vert ^2 =\Vert P_{\tilde{M}^\delta }(\tilde{x}_{k+1})-P_{\tilde{M}^\delta }(x^\dagger )\Vert ^2-\Vert x_k-x^\dagger \Vert ^2 \\&\quad \le \Vert \tilde{x}_{k+1}-x^\dagger \Vert ^2-\Vert x_k-x^\dagger \Vert ^2 =\Vert \tilde{x}_{k+1}-x_k\Vert ^2+2\langle \tilde{x}_{k+1}-x_k,x_k-x^\dagger \rangle \\&\quad =\mu _k^2\Vert \nabla J^\delta (x_k)\Vert ^2-2 \mu _k\langle \nabla J^\delta (x_k),x_k-x^\dagger \rangle . \end{aligned}$$

This can be further estimated under a monotonicity condition on $\nabla J^\delta$ (i.e., convexity condition on $J^\delta$)

18 $$\begin{aligned} \langle \nabla J^\delta (x)- \nabla J^\delta (x^\dagger ),x-x^\dagger \rangle \ge \gamma \Vert \nabla J^\delta (x)\Vert ^2 \quad \text{ for } \text{ all }\; x\in \tilde{M}^\delta \end{aligned}$$

(which for $\gamma =0$ follows from convexity of $J^\delta$, i.e., monotonicity of $\nabla J^\delta$) and assuming approximate stationarity

19 $$\begin{aligned} \langle \nabla J^\delta (x^\dagger ),x-x^\dagger \rangle \ge -\eta (\delta ) \quad \text{ for } \text{ all }\; x\in \tilde{M}^\delta , \end{aligned}$$

Using (18), (19), we get from (17), that for all $k\le k_*-1$ with $k_*$ defined by

20 $$\begin{aligned} k_*=k_*(\delta )=\min \{k: \Vert \nabla J^\delta (x_k)\Vert ^2\le \tau \eta (\delta )\} \end{aligned}$$

the estimate

21 $$\begin{aligned} &\Vert x_{k+1}-x^\dagger \Vert ^2-\Vert x_k-x^\dagger \Vert ^2\\&\quad \le \mu _k^2\Vert \nabla J^\delta (x_k)\Vert ^2-2\mu _k\langle \nabla J^\delta (x_k)-\nabla J^\delta (x^\dagger )\rangle +2\mu _k\eta (\delta )\\&\quad \le -\mu _k\left( 2-\tfrac{\mu _k}{\gamma }-\tfrac{2}{\tau \gamma }\right) \langle \nabla J^\delta (x_k)- \nabla J^\delta (x^\dagger ),x_k-x^\dagger \rangle \\&\quad \le -\mu _k\left( 2\gamma -\mu _k-\tfrac{2}{\tau }\right) \Vert \nabla J^\delta (x_k)\Vert ^2 \le 0 \end{aligned}$$

for $\tau >\frac{1}{\gamma }$, $0<\underline{\mu }\le \mu _k\le \bar{\mu }<2(\gamma -\frac{1}{\tau })$ holds. Hence we get summability

22 $$\begin{aligned}&\sum _{k=0}^{k_*} \langle \nabla J^\delta (x_k)- \nabla J^\delta (x^\dagger ),x_k-x^\dagger \rangle \le \frac{1}{\underline{\mu }\left( 2-\frac{\bar{\mu }}{\gamma }-\tfrac{2}{\tau \gamma }\right) }\Vert x_0-x^\dagger \Vert ^2.\\&\sum _{k=0}^{k_*}\Vert \nabla J^\delta (x_k)\Vert ^2 \le \frac{1}{\underline{\mu }\left( 2\gamma -\bar{\mu }-\tfrac{2}{\tau }\right) } \Vert x_0-x^\dagger \Vert ^2 \end{aligned}$$

Alternatively, we can further estimate (17) under a condition following from (18), (19) and comprising both convexity and approximate stationarity

23 $$\begin{aligned} \langle \nabla J^\delta (x),x-x^\dagger \rangle \ge \gamma \Vert \nabla J^\delta (x)\Vert ^2-\eta (\delta ) \quad \text{ for } \text{ all }\; x\in \tilde{M}^\delta \end{aligned}$$

which for $k\le k_*-1$ implies

$$\begin{aligned} (\gamma \tau 
-1)\eta (\delta )\le \langle \nabla J^\delta (x_k),x_k-x^\dagger \rangle , \end{aligned}$$

as well as

24 $$\begin{aligned} \left( 1+\tfrac{1}{\gamma \tau -1}\right) \langle \nabla J^\delta (x_k),x_k-x^\dagger \rangle \ge \gamma \Vert \nabla J^\delta (x_k)\Vert ^2. \end{aligned}$$

Using (23)–(24) we get from (17)

25 $$\begin{aligned} &\Vert x_{k+1}-x^\dagger \Vert ^2-\Vert x_k-x^\dagger \Vert ^2 \le -\mu _k\left( 2-\tfrac{\mu _k}{\gamma }\left( 1+\tfrac{1}{\gamma \tau -1}\right) \right) \langle \nabla J^\delta (x_k),x_k-x^\dagger \rangle \\&\quad \le -\mu _k\left( \tfrac{2\gamma }{1+\tfrac{1}{\gamma \tau -1}}-\mu _k\right) \Vert \nabla J^\delta (x_k)\Vert ^2 \le 0 \end{aligned}$$

for $\tau >\frac{1}{\gamma }$, $0<\underline{\mu }\le \mu _k\le \bar{\mu } < \tfrac{2\gamma }{1+\tfrac{1}{\gamma \tau -1}}$, hence summability

$$\begin{aligned} \sum _{k=0}^{k_*} \langle \nabla J^\delta (x_k),x_k-x^\dagger \rangle \le \frac{1}{\underline{\mu }\left( 2-\tfrac{\bar{\mu }}{\gamma }\left( 1+\tfrac{1}{\gamma \tau -1}\right) \right) } \Vert x_0-x^\dagger \Vert ^2. \end{aligned}$$

which via (24) also implies summability of $\Vert \nabla J^\delta {(x_k)} \Vert ^2$.

26 $$\begin{aligned} \sum _{k=0}^{k_*} \Vert \nabla J^\delta {(x_k)} \Vert ^2 \le \left( 1+\tfrac{1}{\gamma \tau -1}\right) \frac{1}{\gamma \underline{\mu }\left( 2-\tfrac{\bar{\mu }}{\gamma }\left( 1+\tfrac{1}{\gamma \tau -1}\right) \right) } \Vert x_0-x^\dagger \Vert ^2. \end{aligned}$$

The estimates (22), (26) imply convergence of the gradient to zero as $k\rightarrow \infty$ in the noise free case and finiteness of the stopping index $k_*$ in case of noisy data. In the noiseless case $\delta =0$ Opial’s Lemma (Lemma 2 in the Appendix) with $S=\{x^*\in X:\forall x\in M:\,\langle \nabla J(x^*),x-x^*\rangle \ge 0\}$, due to monotonicity of $\Vert x_k-x^*\Vert$ and the Bolzano–Weierstrass Theorem, implies weak convergence of $x_k$ as $k\rightarrow \infty$ to a stationary point. In case of noisy data, one could think of applying the continuous version of Opial’s Lemma (Lemma 3 in the Appendix) with $t:=\frac{1}{\delta }$, $x(t):=x_{k_*(\delta )}$. However, we do not have monotonicity of the final iterates $x_{k_*(\delta )}$ as a function of $\delta$. Still, in case of uniqueness, that is, if *S* is a singleton $S=\{x^\dagger \}$, then boundedness of the sequence $\Vert x_{k_*(\delta )}-x^*\Vert$ by $\Vert x_0-x^*\Vert$ together with a subsequence-subsequence argument yields its weak convergence of $x_{k_*(\delta )}$ to $x^\dagger$ as $\delta \rightarrow 0$.

For this purpose, we have to impose certain continuity assumptions on the cost function and the constrains, namely

27 $$\begin{aligned}&\text{ For } \text{ any } \text{ sequence } (z_n)_{n\in \mathbb {N}}\subseteq X, \; (\delta _n)_{n\in \mathbb {N}}\in (0,\bar{\delta }], \; \delta _n\rightarrow 0 \text{ as } n\rightarrow \infty\\&\quad \Bigl (\forall n\in \mathbb {N}: \, z_n\in \tilde{M}^{\delta _n} \text{ and } z_n \rightharpoonup z \text{ and } \nabla J^{\delta _n}(z_n)\rightarrow 0\Bigr )\\&\quad \Rightarrow \Bigl (z\in M \text{ and } \forall x\in M:\,\langle \nabla J(z),x-z\rangle \ge 0\Bigr ) \end{aligned}$$

which in the noiseless case becomes

28 $$\begin{aligned} &\text{ For } \text{ any } \text{ sequence } (z_n)_{n\in \mathbb {N}}\subseteq X\\&\quad \Bigl (\forall n\in \mathbb {N}: z_n\in M \text{ and } z_n \rightharpoonup z \text{ and } \nabla J(z_n)\rightarrow 0\Bigr ) \\&\quad \Rightarrow \Bigl (z\in M \text{ and } \forall x\in M:\,\langle \nabla J(z),x-z\rangle \ge 0\Bigr ) \end{aligned}$$

### Proposition 1

*Let* (8), (11), (23) *hold, and let the sequence of iterates* $x_k$ *be defined by* (12) *with* $k_*$ *defined by* (20).

(i) *In the noiseless case* $\delta =0$, *we assume that* *M* *and* $\nabla J$ *satisfy* (28). *Then the sequence* $(x_k)_{k\in \mathbb {N}}$ *converges weakly to a solution* $x^*\in M$ *of the first order optimality condition* (7) *as* $k\rightarrow \infty$.
(ii) *In the noisy case* $\delta >0$, *we assume that* (10), (19), *and* (27) *hold*. *Then the family* $(x_{k_*(\delta )})_{\delta \in (0,\bar{\delta }]}$ *converges weakly subsequentially to a stationary point* $x^\dagger$ *according to* (7) *as* $\delta \rightarrow 0$. *If this stationary point is unique, then the whole sequence converges weakly to* $x^\dagger$.

*The same assertions hold with stationarity* (7) (*with* (27)) *replaced by*

- *minimality, i.e.,* $x^\dagger$ (*and* *z*) $\in \text{ argmin }\{J(x):x\in M\}$ *or by*
- $$\Vert \nabla J(x^\dagger )\Vert =0$$ (*and* $$\Vert \nabla J(z)\Vert =0$$).

Note that cases (a), (b) make uniqueness easier than (7).

### Remark 1

Strong convergence can be shown for the modified projected Landweber method from [5, Section 3.2]. However, this requires a source condition to hold.

### Remark 2

Let us finally comment on the convexity condition (23) and the continuity conditions (27), (28).

In the special case $J^\delta (x)= \tfrac{1}{2}\Vert F(x)-y^\delta \Vert ^2$ cf. (6), condition (23) becomes

29 $$\begin{aligned} \langle F(x)-y^\delta ,F'(x)(x-x^\dagger )\rangle \ge \gamma \Vert F'(x)^*(F(x)-y^\delta )\Vert ^2-\eta (\delta ) \end{aligned}$$

which follows, e.g., from

30 $$\begin{aligned}&\Vert F'(x)\Vert \le 1\quad \text{ and } \\&\quad \langle F(x)-F(x^\dagger )-F'(x)(x-x^\dagger ),F(x)-y^\delta \rangle \le (1-\gamma -\kappa )\Vert F(x)-y^\delta \Vert ^2\\&\quad {x\in \mathcal {D}(F)} \end{aligned}$$

with $\Vert F(x^\dagger )-y^\delta \Vert ^2\le 4\kappa \eta (\delta )$. Here $\mathcal {D}(F)$ does not necessarily need to be the maximal domain of *F* and therefore can be chosen to be a sufficiently small closed ball (hence weakly closed) to enable—together with an appropriate scaling—the uniform boundedness condition on $F'$ in (30). Condition (30) is closely related to the usual normalization and tangential cone conditions for Landweber iteration, see, e.g., [9, 23]. It is, e.g., satisfied for linear *F* as well as for some specific coefficient identification problems, see, e.g., [9] for the reduced setting, [14] for the all-at-once setting, and [20] for some time dependent problems in both reduced and all-at-once formulation.

In the same case (6), with $\tilde{M}^\delta :=\mathcal {D}(F)$, condition (27) can be verified under usual assumptions on *F* cf. e.g., [6, 7] as well: We assume existence of a constant *K* such that

31 $$\begin{aligned}&\Vert F'(x)\Vert \le K \quad x\in \mathcal {D}(F)\quad \text{ and } \\&\quad \Bigl (z_n \rightharpoonup z\;\text{ and }\; F'(z_n)^*(F(z_n)-y)\rightarrow 0\Bigr ) \Rightarrow \Bigl (z\in \mathcal {D}(F)\;\text{ and }\; F'(z)^*(F(z)-y)=0\Bigr ), \end{aligned}$$

where the latter is supposed to hold for any sequence $(z_n)_{n\in \mathbb {N}}\subseteq \mathcal {D}(F)$ and means weak sequential closedness of $x\mapsto F'(x)^*(F(x)-y)$ at zero value. The prerequisite of (27) in this setting reads $z_n \rightharpoonup z \text{ and } F'(z_n)^*(F(z_n)-y^{\delta _n})\rightarrow 0$, and due to $F'(z_n)^*(F(z_n)-y^{\delta _n})=F'(z_n)^*(F(z_n)-y) +F'(z_n)^*(y-y^{\delta _n})$, under these assumptions implies $\Vert F'(z_n)^*(y-y^{\delta _n})\Vert \le K \Vert y-y^{\delta _n}\Vert \rightarrow 0$, hence $F'(z_n)^*(F(z_n)-y)\rightarrow 0$, and therefore $F'(z)^*(F(z)-y)=0$, from which $\langle \nabla J(z),x-z\rangle = \langle \nabla F'(z)^*(F(z)-y),x-z\rangle \ge 0$ trivially follows.

## An SQP type constrained Newton method

A quadratic approximation of the cost function combined with a Tikhonov type additive regularization term yields the iteration scheme

32 $$\begin{aligned}&x_{k+1}\in X_{k+1}(\alpha ):=\text{ argmin}_{x\in \tilde{M}^\delta } Q_k^\delta (x) + \alpha _k\mathcal {R}(x)\\&\quad \text{ where }\;Q_k^\delta (x)=J^\delta (x_k)+G^\delta (x_k)(x-x_k)+\tfrac{1}{2} H^\delta (x_k)(x-x_k)^2 \end{aligned}$$

with

33 $$\begin{aligned}&G^\delta (x_k):X\rightarrow \mathbb {R}\;\text{ linear }, \quad H^\delta (x_k):X^2\rightarrow \mathbb {R}\;\text{ bilinear },\\&\mathcal {R}:X\rightarrow [0,\infty ]\;\text{ proper } \text{ with } \text{ domain }\;\text{ dom }(\mathcal {R})\supseteq \bigcup _{\delta \in (0,\bar{\delta })} \tilde{M}^\delta \cup M \end{aligned}$$

where $G^\delta$ and $H^\delta$ should be viewed as (approximations to) the gradient and Hessian of *J*, $G^\delta (x_k)\approx {J^\delta }'(x_k)$, $H^\delta (x_k)\approx {J^\delta }''(x_k)$, and $\mathcal {R}$ is a regularization functional. Since we do not necessarily neglect terms in ${J^\delta }''(x_k)$, this differs from the iteratively regularized Gauss–Newton method IRGNM studied, e.g., in [2, 17, 22], cf. (35) below.

As opposed to Landweber iteration, where each step on its own is stable, for Newton’s method one has to regularize in each step due to unboundedness of the inverse of the Hessian. To allow the bias due to regularization to vanish as the Newton iteration proceeds, we choose the sequence of regularization parameters $\alpha _k$ such that it tends to zero as $k\rightarrow \infty$.

Here *X* is a general Banach space.

To guarantee existence of minimizers, besides (33) we will make the following assumption

### Assumption 1

For some topology $\mathcal {T}_0$ on *X*

- for all $r\ge \mathcal {R}(x^\dagger )$, the sublevel set $\tilde{M}^\delta _r:=\{x\in M^\delta : \mathcal {R}(x)\le r\}$ is $\mathcal {T}_0$ compact.
- the mapping $Q_k^\delta +\alpha _k\mathcal {R}$ is $\mathcal {T}_0$ lower semicontinuous.

Uniqueness of a minimizer of (32) will not necessarily hold; the sequence $(x_k)_{k\in \{1,\ldots ,k_*\}}$ will therefore be defined by an arbitrary selection of minimizers of (32).

The overall iteration is stopped according to the discrepancy principle

34 $$\begin{aligned} k_*=k_*(\delta )=\min \{k: J^\delta (x_k)\le \tau \eta (\delta )\} \end{aligned}$$

for some constant $\tau >1$.

As far as the sequence of regularization parameters $\alpha _k$ is concerned, we will choose it a priori or a posteriori, see (37), (44) below.

A special case of this with

35 $$\begin{aligned} J^\delta (x)&=\frac{1}{2}\Vert F(x)-y^\delta \Vert ^2, \quad G^\delta (x)h={J^\delta }'(x)h=\langle F(x)-y^\delta , F'(x)h\rangle , \\ {H^\delta }(x)(h,\ell )&=\langle F'(x)h, F'(x)\ell \rangle \end{aligned}$$

(note that $H^\delta$ in general does not coincide with the Hessian of $J^\delta$, since the term containing $F''$ is skipped ) in Hilbert space is the iteratively regularized Gauss–Newton method for the operator equation formulation (3) of the inverse problem, see, e.g., [2, 17, 22].

Another special case we will consider is the quadratic one

36 $$\begin{aligned} J^\delta (x)=f^\delta +\bar{G}^\delta x + \tfrac{1}{2} \bar{H}^\delta x^2, \quad G^\delta (x)h=\bar{G}^\delta h, \quad {H^\delta }(x)(h,\ell )=\bar{H}^\delta (h,\ell ) \end{aligned}$$

with $f^\delta \in X$, $\bar{G}^\delta \in L(X,\mathbb {R})=X^*$, $\bar{H}^\delta \in L(X^2,\mathbb {R})$, where trivially $Q_k^\delta$ coincides with $J^\delta$.

To provide a convergence analysis, we start with the case of an a priori choice of $\alpha _k$

37 $$\begin{aligned} \alpha _k=\alpha _0\theta ^k \end{aligned}$$

for some $\theta \in (0,1)$ and make, among others, the following assumption.

### Assumption 2

For some topology $\mathcal {T}$ on *X*,

- the sublevel set $\{x\in \bigcup _{\delta \in (0,\bar{\delta })}\tilde{M}^\delta : \mathcal {R}(x)\le R\} = \bigcup _{\delta \in (0,\bar{\delta })}\tilde{M}_R^\delta$ is $\mathcal {T}$ compact, with $R=(1+\tfrac{a-b}{\tau (a-b)-c} \tfrac{\tau b+c}{(a\theta -b)}){\mathcal {R}(x^\dagger )} + \tfrac{a-b}{\tau (a-b)-c} \tfrac{\tau b+c}{\alpha _0} J(x_0)$ with $\tau$ as in (34), $\alpha _0$ as in (37), and *a*, *b*, *c* as in (38);
- *M* is $\mathcal {T}$ closed with respect to the family of sets $(\tilde{M}^\delta )_{\delta \in (0,\bar{\delta })}$ in the following sense:
  $$\begin{aligned}&\text{ For } \text{ any } \text{ sequence } (z_n)_{n\in \mathbb {N}}\subseteq X, \, (\delta _n)_{n\in \mathbb {N}}\in (0,\bar{\delta }], \, \delta _n\rightarrow 0 \text{ as } n\rightarrow \infty \\&\Bigl (\forall n\in \mathbb {N}: z_n\in \tilde{M}^{\delta _n} \text{ and } z_n {\mathop {\longrightarrow }\limits ^{\mathcal {T}}} z \Bigr ) \Rightarrow z\in M \end{aligned}$$
- $$\lim _{\delta \rightarrow 0} \sup _{x\in M^\delta }(J(x)-J^\delta (x))\le 0$$;
- *J* is $\mathcal {T}$ lower semicontinuous.

Comparably to the tangential cone condition in the context of nonlinear Landweber iteration [9] and more recently also the IRGNM [22] we impose a restriction on the nonlinearity/nonconvexity of *J*

38 $$\begin{aligned}&G^\delta (x)(x_+-x^+)+\tfrac{1}{2} H^\delta (x)\Bigl ((x_+-x)^2-(x-x^+)^2\Bigr )\ge a J^\delta (x_+)-bJ^\delta (x)\\&\quad -cJ^\delta (x^+)\quad \text{ for } \text{ all }\; x,x_+\in \tilde{M}^\delta , \, x^+=x^\dagger , \quad \delta \in (0,\bar{\delta }), \end{aligned}$$

with $a>b\ge 0$, $c\ge 0$, A discussion of this assumption can be found in Remark 5 below; in view of Taylor’s Theorem, typical values of the constants would be $a\sim 1$, $b\sim 0$, $c\sim 1$.

### Theorem 1

*Let conditions* (10), (11), (33), (38), *and Assumptions* 1, 2*hold, assume that* $\alpha _k$ *is chosen a priori according to* (37), *and* $k_*$ *is chosen according to the discrepancy principle* (34), *with the following constraints on the constants*

$$\begin{aligned} 1>\theta>\frac{b}{a}, \quad \tau >\frac{c}{a-b}. \end{aligned}$$

*Then*

- *For any* $\delta \in (0,\bar{\delta })$, *and any* $x_0\in \bigcap _{\delta \in (0,\bar{\delta })}\tilde{M}^\delta \,\cap M$,
  - *the iterates* $x_k$ *are well-defined for all* $k\le k_*(\delta )$ *and* $k_*(\delta )$ *is finite*;
  - *for all* $k\in \{1,\ldots , k_*(\delta )\}$ *we have*
    $$\begin{aligned} J^\delta (x_k)\le \tfrac{b}{a} J^\delta (x_{k-1}) + \tfrac{1}{a} \alpha _k{\mathcal {R}(x^\dagger )} + \tfrac{c}{a}\eta ; \end{aligned}$$
  - *for all* $k\in \{1,\ldots , k_*(\delta )\}$ *we have*
    $$\begin{aligned} \mathcal {R}(x_k)\le R \end{aligned}$$
- *As* $\delta \rightarrow 0$, *the final iterates* $x_{k_*(\delta )}$ *tend to a solution of the inverse problem* (4) $\mathcal {T}$ *-subsequentially, i.e., every sequence* $x_{k_*(\delta _j)}$ *with* $\delta _j\rightarrow 0$ *as* $j\rightarrow \infty$ *has a* $\mathcal {T}$ *convergent subsequence and the limit of every* $\mathcal {T}$ *convergent subsequence solves* (4).

### Proof

For any $k\le k_*-1$, existence of a minimizer follows from Assumption 1 by the direct method of calculus of variations. To this end, note that by $x^\dagger \in \tilde{M}^\delta$, implying

$$\begin{aligned} \min _{x\in \tilde{M}^\delta } Q_k(x)+\alpha \mathcal {R}(x)\le Q_k(x^\dagger )+\alpha \mathcal {R}(x^\dagger ), \end{aligned}$$

and the lower bound

$$\begin{aligned} Q_k(x)\ge Q_k(x^\dagger )+ a J^\delta (x)-bJ^\delta (x_k)-c J^\delta (x^\dagger )\ge Q_k(x^\dagger )-bJ^\delta (x_k)-c J^\delta (x^\dagger ), \end{aligned}$$

which yields

$$\begin{aligned} \tilde{M}^\delta _r\supseteq \{x\in \tilde{M}^\delta : Q_k(x)+\alpha \mathcal {R}(x)\le Q_k(x^\dagger )+\alpha \mathcal {R}(x^\dagger )\} \end{aligned}$$

for $r=\mathcal {R}(x^\dagger )+\frac{1}{\alpha }(bJ^\delta (x_k)+cJ^\delta (x^\dagger ))$, it suffices to restrict the search for a minimizer to the set $\tilde{M}^\delta _r$ as defined in Assumption 1.

For a hence existing minimizer $x_{k+1}$, its minimality together with feasibility of $x^\dagger$ for (32) yields

39 $$\begin{aligned}&G^\delta (x_k)(x_{k+1}-x_k)+\tfrac{1}{2} H^\delta (x_k)(x_{k+1}-x_k)^2 + \alpha _k\mathcal {R}(x_{k+1})\\&\quad \le G^\delta (x_k)(x^\dagger -x_k)+\tfrac{1}{2} H^\delta (x_k)(x^\dagger -x_k)^2 + \alpha _k\mathcal {R}(x^\dagger ), \end{aligned}$$

which with (38) implies

40 $$\begin{aligned} a J^\delta (x_{k+1}) + \alpha _k\mathcal {R}(x_{k+1}) \le b J^\delta (x_k) + cJ^\delta (x^\dagger ) +\alpha _k\mathcal {R}(x^\dagger ) \end{aligned}$$

thus, with the a priori choice (37), and (10), abbreviating $J_k=J^\delta (x_k)$, $\mathcal {R}_k=\mathcal {R}(x_k)$, $\mathcal {R}^\dagger =\mathcal {R}(x^\dagger )$

41 $$\begin{aligned} J_{k+1} + \tfrac{\alpha _0}{a}\theta ^k\mathcal {R}_{k+1} \le \tfrac{b}{a} J_k + \tfrac{\alpha _0}{a} \theta ^k\mathcal {R}^\dagger + \tfrac{c}{a}{\eta (\delta )}. \end{aligned}$$

Inductively, with $\mathcal {R}\ge 0$, we conclude that for all $k\le k_*$

42 $$\begin{aligned} J_k&\le \left( \tfrac{b}{a}\right) ^k J_0 + \tfrac{\alpha _0}{a}\mathcal {R}^\dagger \sum _{j=0}^{k-1} \left( \tfrac{b}{a}\right) ^j\theta ^{k-1-j}+ \tfrac{c}{a}{\eta (\delta )} \sum _{j=0}^{k-1} \left( \tfrac{b}{a}\right) ^j\\&\le \left( \tfrac{b}{a}\right) ^k J_0 + \tfrac{\alpha _0}{a\theta -b}\mathcal {R}^\dagger \theta ^k+ \tfrac{c}{a-b}{\eta (\delta )}. \end{aligned}$$

Using the minimality of $k_*$ according to (34), we get, for all $k\le k_*-1$, that ${\eta (\delta )}\le \frac{J_k}{\tau }$ and therefore, together with (42)

$$\begin{aligned} \left( 1-\tfrac{c}{\tau (a-b)}\right) J_k \le \left( \tfrac{b}{a}\right) ^k J_0 + \tfrac{\alpha _0}{a\theta -b}\mathcal {R}^\dagger \theta ^k. \end{aligned}$$

Inserting this back into (41) with $J^\delta \ge 0$, after multiplication by $\frac{a}{\alpha _k}$ and again using (34) yields

43 $$\begin{aligned} \mathcal {R}_{k+1}&\le \tfrac{b}{\alpha _k} J_k + \mathcal {R}^\dagger +\tfrac{c}{\alpha _k} \tfrac{J_k}{\tau } \le \mathcal {R}^\dagger +\tfrac{\tau b+c}{\tau \alpha _0} \theta ^{-k} J_k\\&\le \mathcal {R}^\dagger + \tfrac{a-b}{\tau (a-b)-c} \tfrac{\tau b+c}{\alpha _0} \left( \left( \tfrac{b}{a\theta }\right) ^k J_0 + \tfrac{\alpha _0}{a\theta -b}\mathcal {R}^\dagger \right) = R \end{aligned}$$

for all $$k\le k_*-1$$.

From (42), which holds for all $k\le k_*$ and $\tau >\tfrac{c}{a-b}$, as well as $\tfrac{b}{a}<\theta$, we conclude that the stopping index according to (34) is reached after finitely many, namely at most $\frac{\log \left( \left( \tau -\tfrac{c}{a-b}\right) {\eta (\delta )}\right) -\log \left( J_0+\tfrac{\alpha _0}{a\theta -b}\mathcal {R}^\dagger \right) }{\log \theta }$ steps.

Setting $k=k_*-1$ in (43) yields $\mathcal {R}(x_{k_*(\delta )})\le R$, which implies $\mathcal {T}$ convergence of a subsequence $x^j$ of $x_{k_*(\delta )}$ to some $\bar{x}$, which by Assumption 2 lies in *M*.

By definition of $k_*$ and (10) we have $J(x_{k_*(\delta )})\le \tau {\eta (\delta )}(\delta )+J(x_{k_*(\delta )})-J^\delta (x_{k_*(\delta )})\rightarrow 0$ as $\delta \rightarrow 0$; $\mathcal {T}$ lower semicontinuity therefore yields $J(\bar{x})=0$. $\diamondsuit$

We now consider convergence with an a posteriori choice of $\alpha _k$ according to the discrepancy principle type rule (which can also be interpreted as an inexact Newton condition)

44 $$\begin{aligned} \underline{\sigma }\le \sigma _k(\alpha _k):=\frac{Q_k^\delta (X_{k+1}(\alpha _k))}{J^\delta (x_k)}\le \overline{\sigma } \end{aligned}$$

with $0<\underline{\sigma }<\overline{\sigma }<1$; note that in (44), the denominator of $\sigma _k(\alpha _k)$ will be positive and bounded away from zero by $\tau \eta (\delta )$ for all $k\le k_*(\delta )-1$ by (34). In order to obtain well-definedness of $\sigma _k(\alpha )$ as a function of $\alpha$, we will assume that the mapping

$$\begin{aligned} \alpha \mapsto Q_k^\delta (X_{k+1}(\alpha ))\quad \text{ with }\quad X_{k+1}(\alpha )={\text{ argmin }}_{x\in \tilde{M}^\delta }(Q_k^\delta (x)+\alpha \mathcal {R}(x)) \end{aligned}$$

is single valued, which is, e.g., the case if the minimizer of $Q_k^\delta (x)+\alpha \mathcal {R}(x)$ over $\tilde{M}^\delta$ is unique. The latter can be achieved, e.g., by assuming convexity of $Q_k^\delta$—choosing $H^\delta$ as a positive semidefinite approximation of the (not necessarily positive semidefinite) true Hessian ${J^\delta }''$—and strict convexity of $\mathcal {R}$. To numerically determine $\alpha _k$ such that it satisfies (44), some iterative procedure such as the bisection method can be applied. For the a posteriori choice (44) we have to slightly modify the setting to guarantee existence of $\alpha _k$ such that (44) holds. The latter is possible if for some appropriate point $x^*$, the quotient $\frac{Q^\delta _k(x^*)}{J^\delta (x_k)}$ is large enough

45 $$\begin{aligned} \underline{\sigma }<\frac{Q^\delta _k(x^*)}{J^\delta (x_k)} \end{aligned}$$

as we will show below. This leads us to the following case distinction for updating the iterates

$$\begin{aligned}&\text{ If } \text{(45) } \text{ holds, } \text{ choose }\;\alpha _k\;\hbox {according to (44) and}\; x_{k+1}\; \hbox {as in}\; (32)\\&\text{ otherwise } \text{ set }\;x_{k+1}=x^*. \end{aligned}$$

Here $x^*\in \bigcap _{\delta \in (0,\bar{\delta })} \tilde{M}^\delta \cap M$ is a point of attraction of $\mathcal {R}$ in the sense of the following assumption.

### Assumption 3

For some topology $\mathcal {T}_1$ on *X*,

- $\mathcal {R}(x^*)=0$ and for any sequence $(x_j)_{j\in \mathbb {N}}\subseteq X$
  46 $$\begin{aligned} \mathcal {R}(x_j)\rightarrow 0 \Rightarrow x_j {\mathop {\longrightarrow }\limits ^{\mathcal {T}_1}} x^* \end{aligned}$$
- sublevel sets of $\mathcal {R}$ are $\mathcal {T}_1$ compact;
- $\mathcal {R}$ is $\mathcal {T}_1$ lower semicontinuous;
- the mapping $x\mapsto G^\delta (x_k)(x-x_k)+\tfrac{1}{2} H^\delta (x_k)(x-x_k)^2$ is $\mathcal {T}_1$ continuous
- $$\tilde{M}^\delta$$ is $$\mathcal {T}_1$$ closed.

A simple example of a functional $\mathcal {R}$ satisfying this assumption is some power of the norm distance from the a priori guess $x^*$, $\mathcal {R}(x)=\Vert x-x^*\Vert ^p$, for some $p\in [1,\infty )$ along with the weak or weak* topology $\mathcal {T}_1$, provided *X* is reflexive or the dual of a separable space.

### Lemma 1

*The mappings* $\alpha \mapsto \mathcal {R}(x_{k+1}(\alpha ))$ *and* $\alpha \mapsto -Q_k(x_{k+1}(\alpha ))$, *where* $x_{k+1}(\alpha )\in X_{k+1}(\alpha )$ (*cf*. (32)) *are monotonically decreasing*.

*If additionally Assumption* 3*and* (38) *hold, and the mapping* $\alpha \mapsto Q_k(X_{k+1}(\alpha ))$ *is single valued, then the mapping* $\alpha \mapsto \sigma _k(\alpha )$ *is well-defined and continuous on* $(0,\infty )$.

### Proof

For two values $\alpha$, $\tilde{\alpha }$, minimality implies

$$\begin{aligned}&Q_k(x_{k+1}(\alpha ))+\alpha \mathcal {R}(x_{k+1}(\alpha )) \le Q_k(x_{k+1}(\tilde{\alpha }))+\alpha \mathcal {R}(x_{k+1}(\tilde{\alpha }))\\&\quad = Q_k(x_{k+1}(\tilde{\alpha }))+\tilde{\alpha }\mathcal {R}(x_{k+1}(\tilde{\alpha })) +(\alpha -\tilde{\alpha })\mathcal {R}(x_{k+1}(\tilde{\alpha }))\\&\quad \le Q_k(x_{k+1}(\alpha ))+\tilde{\alpha }\mathcal {R}(x_{k+1}(\alpha )) +(\alpha -\tilde{\alpha })\mathcal {R}(x_{k+1}(\tilde{\alpha })) \end{aligned}$$

which implies

$$\begin{aligned} 0\ge (\alpha -\tilde{\alpha })(\mathcal {R}(x_{k+1}(\alpha ))-\mathcal {R}(x_{k+1}(\tilde{\alpha }))). \end{aligned}$$

Hence, $\alpha \mapsto \mathcal {R}(x_{k+1}(\alpha ))$ is monotonically decreasing and

$$\begin{aligned} Q_k(x_{k+1}(\alpha ))-Q_k(x_{k+1}(\tilde{\alpha })) \le \alpha (\mathcal {R}(x_{k+1}(\tilde{\alpha }))-\mathcal {R}(x_{k+1}(\alpha )))\le 0 \text{ for } \alpha \le \tilde{\alpha } \end{aligned}$$

that is, $\alpha \mapsto Q_k(x_{k+1}(\alpha ))$ is monotonically increasing.

To prove continuity of the mapping $\alpha \mapsto Q_k(x_{k+1}(\alpha ))$ under the assumption that this mapping is single valued, consider $\bar{\alpha }>0$ and a sequence $(\alpha _\ell )_{\ell \in \mathbb {N}}$ converging to $\bar{\alpha }>0$. Minimality and (38) yield

$$\begin{aligned} \alpha _\ell \mathcal {R}(x_{k+1}(\alpha _\ell ))&\le Q_k(x^\dagger ) +\alpha _\ell \mathcal {R}(x^\dagger ) - Q_k(x_{k+1}(\alpha _\ell ))\\&\le -a J^\delta (x_{k+1}(\alpha _\ell ))+b J^\delta (x_k)+c J^\delta (x^\dagger )+\alpha _\ell \mathcal {R}(x^\dagger ) \\&\le b J^\delta (x_k)+c J^\delta (x^\dagger )+\alpha _\ell \mathcal {R}(x^\dagger ), \end{aligned}$$

which by strict positivity of $\bar{\alpha }$ implies boundedness of $(\mathcal {R}(x_{k+1}(\alpha _\ell )))_{\ell \in \mathbb {N}}$. By Assumption 3 there exists a $\mathcal {T}_1$ convergent subsequence $(x_{k+1}(\alpha _{\ell _j}))_{j\in \mathbb {N}}$ whose limit $\bar{x}$ lies in $\tilde{M}^\delta$ and even in $X_{k+1}(\alpha )$, due to the fact that $Q_k(x_{k+1}(\alpha _{\ell _j}))\rightarrow Q_k(\bar{x})$ and the estimate

$$\begin{aligned} Q_k(\bar{x}) +\bar{\alpha }\mathcal {R}(\bar{x})&\le \liminf _{j\rightarrow \infty } \Bigl (Q_k(x_{k+1}(\alpha _{\ell _j})) +\alpha _{\ell _j} \mathcal {R}(x_{k+1}(\alpha _{\ell _j}))\Bigr )\\&\le \liminf _{j\rightarrow \infty } \Bigl (Q_k(x_{k+1}(\bar{\alpha })) +\alpha _{\ell _j} \mathcal {R}(x_{k+1}(\bar{\alpha }))\Bigr )\\&= Q_k(x_{k+1}(\bar{\alpha })) +\bar{\alpha } \mathcal {R}(x_{k+1}(\bar{\alpha })). \end{aligned}$$

A subsequence-subsequence argument together with $\mathcal {T}_1$ continuity of $Q_k$ and the assumed single valuedness of the mapping $\alpha \mapsto Q_k(X_{k+1}(\alpha ))$ implies convergence $Q_k(x_{k+1}(\alpha _\ell ))\rightarrow Q_k(x_{k+1}(\bar{\alpha }))$, hence, after division by $J^\delta (x_k)$, convergence $\sigma _k(x_{k+1}(\alpha _\ell ))\rightarrow \sigma _k(x_{k+1}(\bar{\alpha }))$. $\diamondsuit$

To prove convergence of the iterates, we need a slightly stronger condition than (38), namely

47 $$\begin{aligned} \underline{a}J^\delta (x_+)-\underline{b}J^\delta (x)&\le G^\delta (x)(x_+-x)+\tfrac{1}{2} H^\delta (x)(x_+-x)^2\\&\le \overline{a}J^\delta (x_+)-\overline{b}J^\delta (x)\quad \text{ for } \text{ all }\; x,x_+\in \tilde{M}^\delta , \quad \delta \in (0,\bar{\delta }), \end{aligned}$$

with $\underline{a},\underline{b},\overline{a},\overline{b}\ge 0$. Note that (47) implies $(\overline{a}-\underline{a})J^\delta (x_+)+(\underline{b}-\overline{b})J^\delta (x)\ge 0$, hence by nonnegativity of $J^\delta$ and the fact that $J^\delta (x^\dagger )\le \eta$ can get arbitrarily close to zero, $\overline{a}\ge \underline{a}$ and $\underline{b}\ge \overline{b}$. In fact, (47) implies (38) with $a=\underline{a}$, $b=\underline{b}-\overline{b}$, $c=\overline{a}$.

### Theorem 2

*Let conditions* (10), (11), (33), (47), *and Assumptions* 1, 2, 3*hold, assume that* $\alpha _k$ *is chosen a posteriori according to* (44) *if* (45) *holds* (*otherwise set* $x_{k+1}:=x^*$), *and* $k_*$ *is chosen according to the discrepancy principle* (34), *with the following constraints on the constants*

48 $$\begin{aligned} 1+\frac{\bar{a}}{\tau }<\underline{\sigma }+\overline{b}, \quad \overline{\sigma }+\underline{b}<1+\underline{a}. \end{aligned}$$

*Then*

- *For any* $\delta \in (0,\bar{\delta })$, *and any* $x_0\in \bigcap _{\delta \in (0,\bar{\delta })}{\tilde{M}^\delta }$,
  - *the iterates* $x_k$ *are well-defined for all* $k\le k_*(\delta )$ *and* $k_*(\delta )$ *is finite*;
  - *for all* $k\in \{1,\ldots , k_*(\delta )\}$ *and* $q=\frac{\overline{\sigma } -1+\underline{b}}{\underline{a}}<1$ *we have*
    $$\begin{aligned} J^\delta (x_k)\le q J^\delta (x_{k-1}); \end{aligned}$$
  - *for all* $k\in \{1,\ldots , k_*(\delta )\}$ *and* $x^\dagger$ *satisfying* (10) *we have*
    $$\begin{aligned} \mathcal {R}(x_k)\le \mathcal {R}(x^\dagger )\quad \text{ and }\quad x^\dagger \;\text{ solves } \text{(4). } \end{aligned}$$
- *As* $\delta \rightarrow 0$, *the final iterates* $x_{k_*(\delta )}$ *tend to a solution of the inverse problem* (4) $\mathcal {T}$ *-subsequentially, i.e., every sequence* $x_{k_*(\delta _j)}$ *with* $\delta _j\rightarrow 0$ *as* $j\rightarrow \infty$ *has a* $\mathcal {T}$ *convergent subsequence and the limit of every* $\mathcal {T}$ *convergent subsequence solves* (4).

### Proof

Existence of minimizers $x_{k+1}(\alpha )$ of (32) with $\alpha >0$ in place of $\alpha _k$ follows like in the a priori setting of Theorem 1, using the fact that (47) implies (38).

To prove that $\alpha _k$ satisfying (44) exists under condition (45), we first of all verify the upper bound with $\alpha =0$ (which actually does not require (45)). To this end, we make use of minimality (39) and the upper bound in (47) to conclude

$$\begin{aligned} \sigma _k(\alpha )&\le \frac{J^\delta (x_k)+G^\delta (x_k)(x^\dagger -x_k)+\tfrac{1}{2} H^\delta (x_k)(x^\dagger -x_k)^2 + \alpha (\mathcal {R}(x^\dagger )-\mathcal {R}(x_{k+1}(\alpha )))}{J^\delta (x_k)}\\&\le 1-\overline{b}+\bar{a}\frac{J^\delta (x^\dagger )}{J^\delta (x_k)}+\alpha \frac{\mathcal {R}(x^\dagger )-\mathcal {R}(x_{k+1}(\alpha ))}{J^\delta (x_k)}, \end{aligned}$$

so that by (34), for any $k\in \{1,\ldots ,k_*-1\}$

$$\begin{aligned} \lim _{\alpha \searrow 0}\sigma _k(\alpha )\le 1-\overline{b}+\frac{\bar{a}}{\tau }<\underline{\sigma }. \end{aligned}$$

On the other hand, minimality and the fact that $x^*\in \tilde{M}^\delta$ together with the lower bound in (47) and $\mathcal {R}(x^*)=0$ yield

$$\begin{aligned}&\underline{a}J^\delta (x_{k+1}(\alpha ))-\underline{b}J^\delta (x_k) + \alpha \mathcal {R}(x_{k+1}(\alpha ))\\&\quad \le G^\delta (x_k)(x_{k+1}(\alpha )-x_k)+\tfrac{1}{2} H^\delta (x_k)(x_{k+1}(\alpha )-x_k)^2 + \alpha \mathcal {R}(x_{k+1}(\alpha ))\\&\quad \le G^\delta (x_k)(x^*-x_k)+\tfrac{1}{2} H^\delta (x_k)(x^*-x_k)^2 \end{aligned}$$

which by nonnegativity of $\underline{a}J^\delta (x_{k+1}(\alpha ))$ yields

$$\begin{aligned} \mathcal {R}(x_{k+1}(\alpha ))& \le \frac{1}{\alpha }\Bigl (\underline{b}J^\delta (x_k)+G^\delta (x_k)(x^*-x_k)+\tfrac{1}{2} H^\delta (x_k)(x^*-x_k)^2\Bigr )\\ &\rightarrow 0\quad \text{ as }\;\alpha \rightarrow \infty \end{aligned}$$

which by Assumption 3 implies $\mathcal {T}_1$ convergence of $x_{k+1}(\alpha )$ to $x^*$, thus, by (45) $\lim _{\alpha \rightarrow \infty } \sigma _k(\alpha )\ge \underline{\sigma }$. The Intermediate Value Theorem together with continuity of the mapping $\alpha \mapsto \sigma _k(\alpha )$ according to Lemma 1 implies existence of an $\alpha \in (0,\infty )$ such that $\underline{\sigma }\le \sigma _k(\alpha )\le \overline{\sigma }$.

In both cases we get geometric decay of the cost function values: If (45) is satisfied, this follows from the lower bound in (47) and the upper bound in (44)

$$\begin{aligned} J^\delta (x_{k+1})&\le \tfrac{1}{\underline{a}}\left( \underline{b}J^\delta (x_k)+G^\delta (x_k)(x_{k+1}-x_k)+\tfrac{1}{2} H^\delta (x_k)(x_{k+1}-x_k)^2\right) \\&\le \frac{\overline{\sigma } -1+\underline{b}}{\underline{a}} J^\delta (x_k). \end{aligned}$$

Otherwise, negation of (45) and the fact that in that case we set $x_{k+1}=x^*$, together with the lower bound in (47) directly yields

$$\begin{aligned} J^\delta (x_{k+1})&=J^\delta (x^*)\le \tfrac{1}{\underline{a}}\left( \underline{b}J^\delta (x_k)+G^\delta (x_k)(x^*-x_k)+\tfrac{1}{2} H^\delta (x_k)(x^*-x_k)^2\right) \\&\le \frac{\underline{\sigma } -1+\underline{b}}{\underline{a}} J^\delta (x_k). \end{aligned}$$

This implies that $k_*$ is finite, more precisely $k_*\le \frac{\log (\tau \eta )-\log (J^\delta (x_0))}{\log (q)}$.

To establish the bound on $\mathcal {R}(x_{k+1})$, we again employ minimality (39) together with (47), which in case (45) with (44) yields

$$\begin{aligned}&\underline{\sigma }J^\delta (x_k) + \alpha _k\mathcal {R}(x_{k+1})\\&\quad \le J^\delta (x_k)+G^\delta (x_k)(x_{k+1}-x_k)+\tfrac{1}{2} H^\delta (x_k)(x_{k+1}-x_k)^2 + \alpha _k\mathcal {R}(x_{k+1})\\&\quad \le J^\delta (x_k)+G^\delta (x_k)(x^\dagger -x_k)+\tfrac{1}{2} H^\delta (x_k)(x^\dagger -x_k)^2 + \alpha _k\mathcal {R}(x^\dagger )\\&\quad \le \overline{a}J^\delta (x^\dagger ) +(1-\overline{b})J^\delta (x_k)+\alpha _k\mathcal {R}(x^\dagger ), \end{aligned}$$

hence, due to (34), $\tau (\overline{b}+\underline{\sigma }-1)\ge \overline{a}$,

$$\begin{aligned} \mathcal {R}(x_{k+1})\le \mathcal {R}(x^\dagger )+\frac{1}{\alpha _k} \Bigl (\overline{a}J^\delta (x^\dagger )-(\overline{b}+\underline{\sigma }-1) J^\delta (x_k)\Bigr )\le \mathcal {R}(x^\dagger ). \end{aligned}$$

If (45) fails to hold then we set $x_{k+1}=x^*$, hence get $\mathcal {R}(x_{k+1})=0$.

The rest of the proof is the same as for Theorem 1. $\diamondsuit$

### Remark 3

The computational cost for each Newton step (as compared to a gradient method) is determined by the effort spent on evaluating the action of the Hessian (approximation) $H^\delta (x_k)$ on a vector and on solving systems with $H^\delta (x_k)$ as a system matrix—again usually based on iterative methods and matrix-vector products. For example, when usung the exact Hessian as $H^\delta (x_k)$ in a reduced formulation of the inverse problem, each matrix-vector product amounts to numerically solving a possibly large number of linearized versions of the PDE model; this effort can be reduced to just one PDE solve by means of adjoint methods. On the other hand, setting $H^\delta (x_k)$ just to a multiple of the identity, one can even end up with a plain gradient method. However, in view of Taylor’s Theorem one is more likely to satisfy condition (38) with a better Hessian approximation. In practice, a limited memory BFGS method can probably be viewed as standard and only needs gradient information; note however, that for the cost functions in our numerical examples it would also be possible to find analytical expressions for the Hessian, cf., e.g., (58), whose action on a vector can be evaluated by means of, e.g., a finite element method.

The advantages of method (32) lie in its versatility: Besides the various options of choosing $H^\delta (x_k)$, it also works in general Banach spaces and with quite general regularization functionals $\mathcal {R}$. In case of a quadratic functional $\mathcal {R}$, note that as opposed to the IRGNM considered, e.g., in [17, 22, 29], where depending on the choice of the data misfit term, the cost function can become nonlinear, we always deal with a quadratic overall cost function here.

### Remark 4

Note that the conditions (48) on the constants can be satisfied by choosing $\tau$ sufficiently large and $\underline{\sigma }<\overline{\sigma }$ in an appropriate way, provided the constants in (47) satisfy

$$\begin{aligned} \underline{b} <\underline{a}+\overline{b}, \end{aligned}$$

since then we can choose $\underline{\sigma },\overline{\sigma }$ to satisfy $1-\overline{b}<\underline{\sigma }<\overline{\sigma }<1+\underline{a}-\underline{b}$, so that (48) can be achieved by making $\tau$ large enough.

### Remark 5

Condition (47) is motivated by the fact that

$$\begin{aligned} G^\delta (x)(x_+-x)+\tfrac{1}{2} H^\delta (x)(x_+-x)^2\approx J^\delta (x_+)-J^\delta (x), \end{aligned}$$

with equality in case of a quadratic functional $J^\delta$ (36) from which (again using nonnegativity of $J^\delta$) we expect values $\underline{a}\le 1$, $\underline{b}\ge 1$, $\overline{a}\ge 1$, $\overline{b}\le 1$ where these constants can be chosen the closer to one the closer $J^\delta$ is to a quadratic functional. Also note that (47) holds with $\underline{a}=\underline{b}=\overline{a}=\overline{b}$ in the quadratic case (36) independently of the definiteness of the Hessian, so does not necessarily relate to convexity of $J^\delta$. Indeed, while nonnegativity of the Hessian would be enforced by assuming $J^\delta \ge 0$ on all of *X*, we only assume this to hold on $\tilde{M}^\delta$ cf. (11).

A sufficient condition for (47) (with $\underline{a}=1-\tilde{c}$, $\underline{b}=1+\tilde{c}$, $\overline{a}=1+\tilde{c}$, $\overline{b}=1-\tilde{c}$) is

49 $$\begin{aligned}&\tilde{c}(J^\delta (x_+)+J^\delta (x)) \ge |J^\delta (x_+)-J^\delta (x)-G^\delta (x)(x_+-x)-\tfrac{1}{2} H^\delta (x)(x_+-x)^2|\\&\quad \text{ for } \text{ all }\; x,x_+\in \tilde{M}^\delta , \quad \delta \in (0,\bar{\delta }), \end{aligned}$$

which, in its turn is implied by the weak tangential cone condition in the Hilbert space least squares setting (35)

50 $$\begin{aligned}&|\langle F(x_+)-F(x)-F'(x)(x_+-x),F(x)-y^\delta \rangle | \le c_{tc}\Vert F(x_+)-F(x)\Vert \, \Vert F(x)-y^\delta \Vert \\&\quad \text{ for } \text{ all }\; x,x_+\in \tilde{M}^\delta , \quad \delta \in (0,\bar{\delta }), \end{aligned}$$

with $\tilde{c}=(1+\sqrt{2})c_{tc}$; cf. (30). This can be seen by using the fact that the left hand side in (50) just equals the left hand side in (49) with (35), and by estimating the right hand side with $\alpha :=\Vert F(x_+)-y^\delta \Vert$, $\beta :=\Vert F(x)-y^\delta \Vert$ as follows

$$\begin{aligned} \Vert F(x_+)-F(x)\Vert \, \Vert F(x)-y^\delta \Vert &\le (\alpha +\beta )\beta \le \frac{1+\sqrt{2}}{2}(\alpha ^2+\beta ^2)\\ &= (1+\sqrt{2})(J^\delta (x_+)+J^\delta (x)). \end{aligned}$$

Condition (50) with $x_+=x^\dagger$ is also sufficient for condition (29) from the previous section with $\gamma = 1-c_{tc}-\kappa$ provided $(1+c_{tc})\Vert F(x)-y^\delta \Vert \le 2\sqrt{\kappa \eta (\delta )}$ and $\Vert F'(x)\Vert \le 1$ as the estimate

$$\begin{aligned}&\langle F'(x)(x-x^\dagger ),F(x)-y^\delta \rangle \ge \langle F(x)-F(x^\dagger ),F(x)-y^\delta \rangle \\&\qquad - c_{tc}\Vert F(x)-F(x^\dagger )\Vert \, \Vert F(x)-y^\delta \Vert \\&\quad = \Vert F(x)-y^\delta \Vert ^2 - \langle F(x^\dagger )-y^\delta ,F(x)-y^\delta \rangle \\&\qquad - c_{tc}(\Vert F(x)-y^\delta -(F(x^\dagger )-y^\delta )\Vert \, \Vert F(x)-y^\delta \Vert \\&\quad \ge (1-c_{tc}) \Vert F(x)-y^\delta \Vert ^2 -(1+c_{tc}) \Vert F(x^\dagger )-y^\delta \Vert \,\Vert F(x)-y^\delta \Vert \\&\quad \ge (1-c_{tc}-\kappa ) \Vert F(x)-y^\delta \Vert ^2 -\frac{(1+c_{tc})^2}{4\kappa } \Vert F(x^\dagger )-y^\delta \Vert ^2 \end{aligned}$$

following from (50) with the triangle inequality and Young’s inequality shows.

In order to further relate the assumptions (38), (47) made for Newton’s method with those (18), (23) for the projected gradient method, we will now point out that actually also the sufficient condition (49) involves some convexity.

For this purpose we consider the noise free case $\delta =0$ for simplicity of exposition and use the fact that for $n\in \mathbb {N}_0$, a functional $J\in C^n(X)$ and elements $x,\tilde{x},h\in X$, the identity

$$\begin{aligned} (J^{(n-1)}(\tilde{x})-J^{(n-1)}(x))[h^{n-1}] = \int _0^1 J^{(n)}[x+\theta (\tilde{x}-x))[\tilde{x}-x,h^{n-1}]\, d\theta \end{aligned}$$

holds. Thus we can rewrite the left hand sides of the nonlinearity conditions (18), (49) as

51 $$\begin{aligned} \langle \nabla J(x)- \nabla J(x^\dagger ),x-x^\dagger \rangle&=J'(x)- J'(x^\dagger )[x-x^\dagger ]\\&=\int _0^1 J''(x^\dagger +\theta (x-x^\dagger ))[(x-x^\dagger )]^2\, d\theta , \end{aligned}$$

and, with $$J(x^\dagger )=0$$

$$\begin{aligned} J(x_+)+J(x) = (J(x_+)-J(x^\dagger ))+(J(x)-J(x^\dagger )) \end{aligned}$$

where, assuming $J'(x^\dagger )=0$ (as is the case in the examples from Sect. 4)

52 $$\begin{aligned} J(x)-J(x^\dagger )&= \int _0^1 \Bigl (J'(x^\dagger +\theta (x-x^\dagger ))-J'(x^\dagger )\Bigr )[x-x^\dagger ]\, d\theta \\&=\int _0^1 \int _0^1 \theta J''(x^\dagger +\theta \sigma (x-x^\dagger ))[(x-x^\dagger )^2]\,d\sigma \, d\theta \end{aligned}$$

and likewise for *x* replaced by $x_+$. Similarly, using the identities $\int _0^1\, d\theta =1$, $\int _0^1\int _0^1\theta \,d\sigma \, d\theta =\frac{1}{2}$, one sees that for the right hand side in (49) with $G:=J'$, $H:=J''$, the identity

$$\begin{aligned}&J(x_+)-J(x)-J'(x)(x_+-x)-\tfrac{1}{2} J''(x)(x_+-x)^2 \\&\quad = \int _0^1\int _0^1\int _0^1\theta ^2\sigma J'''(x^\dagger +\theta \sigma \rho (x-x^\dagger ))[(x-x^\dagger )^3]\, d\rho \, d\sigma \, d\theta \end{aligned}$$

holds.

Since the left hand sides in (18), (49) both have to be nonnegative (in some uniform sense) we see from (51) and (52) (and setting $x_+=x^\dagger$ in (49) to see necessity) that $J''$ needs to be positive definite (in some uniform sense) in order for (18), (49) to hold. This amounts to a convexity condition on *J*.

### Remark 6

Alternatively to (32) one could consider the projected versions (based on unconstrained minimization)

53 $$\begin{aligned} \tilde{x}_{k+1}&\in {\text{ argmin }}_{x\in X} J^\delta (x_k)+G(x_k)(x-x_k)+\tfrac{1}{2} H(x_k)(x-x_k)^2 + \alpha _k\mathcal {R}(x)\\ x_{k+1}&= {\text{ Proj }}_{\tilde{M}^\delta }(\tilde{x}_{k+1}) \end{aligned}$$

see [18] which, however, analogously to the projected Landweber iteration from [5, Section 3.2] only converges under a sufficiently strong source condition.

## Application in diffusion/impedance identification

Following the seminal idea from [24] we consider variational formulations of the problem of identifying the spatially varying parameter $\sigma$ in the elliptic PDE

54 $$\begin{aligned} \nabla \cdot (\sigma \nabla \phi )=0\quad \text{ in }\;\varOmega \end{aligned}$$

from observations of $\phi$. Depending on what kind of observations we consider, this problem arises in several applications that we will consider here, namely

- in classical electrical impedance tomography EIT, where it is known as Calderon’s problem and $\sigma$ plays the role of an electrical conductivity,
- in impedance acoustic tomography IAT, a novel hybrid imaging method, again for reconstructing $\sigma$ as a conductivity;
- but also as a simplified version of the inverse groundwater filtration problem GWF of recovering the diffusion coefficient $\sigma$ in an aquifer.

Although we will finally be only able to verify the crucial conditions (18), (49) for GWF, we stick to the electromagnetic context notation wise, since in our numerical experiments we will focus on a version of EIT that is known as impedance acoustic tomography IAT, see, e.g., [34]. In Sect. 5 we will also allow for experiments with several excitations (and corresponding measurements), hence consider

$$\begin{aligned} \nabla \cdot (\sigma \nabla \phi _i)=0\quad \text{ in }\;\varOmega , \quad i\in \{1,\ldots ,I\}. \end{aligned}$$

However for simplicity of notation, we will focus on the case $I=1$, i.e., (54), in this section. The observations are, depending on the application

$$\begin{aligned} v&= \phi \vert _{\partial \varOmega }\;\text{(the } \text{ voltage } \text{ at } \text{ the } \text{ boundary) } \text{ in } \text{ EIT, }\\ \mathcal {H}&=\sigma |\nabla \phi |^2\;\text{(the } \text{ power } \text{ density) } \text{ in } \text{ IAT, } \\ p&= \phi \text{ or } g = \nabla \phi \;\text{(the } \text{ piezometric } \text{ head } \text{ or } \text{ its } \text{ gradient) } \text{ in } \text{ GWF, } \end{aligned}$$

where for EIT and IAT we will consider the more realistic complete electrode model in Sect. 5. Concerning GWF, measurements are actually done on the piezometric head itself, however this allows to recover an approximation of its gradient by means of regularized numerical differentiation, see, e.g. [10] and the references therein.

Regularization is here only introduced via imposing simple bound constraints $\sigma \in [\underline{\sigma },\overline{\sigma }]$, that is, Ivanov regularization. Besides the availability of efficient optimization methods for solving such problems, see e.g. [11] and the references therein and the straightforward use of known physical bounds, this has the advantage of leading to piecewise constant solutions, a fact that can be explained by some bang-bang principle. This is relevant when identifying inclusions in a homogeneous background, which is the case that we focus on in our numerical experiments.

Considering a smooth and simply connected bounded domain $\varOmega \subseteq \mathbb {R}^2$ and using the vector fields $\mathbf {E}$ (the electric field), $\mathbf {J}$ (the current density), where $$\nabla =\left( \begin{array}{c}\partial _1\\ \partial _2\end{array}\right)$$, $$\nabla ^\bot =\left( \begin{array}{c}-\partial _2\\ \partial _1\end{array}\right)$$ we can equivalently rephrase (54) as

$$\begin{aligned} \sigma \mathbf {E}= \mathbf {J}, \quad \mathbf {E}=\nabla \phi , \quad \mathbf {J}=\nabla ^\bot \psi , \end{aligned}$$

for some potential $\psi$ (note that we are using the opposite sign convention as compared to the usual engineering notation). The cost function part pertaining to this model is, analogously to [24], therefore often called the Kohn–Vogelius functional

55 $$\begin{aligned} J_{mod}^{KV}(\sigma ,\mathbf {E},\mathbf {J})=\tfrac{1}{2}\int _\varOmega \left| \sqrt{\sigma }\mathbf {E}-\tfrac{1}{\sqrt{\sigma }}\mathbf {J}\right| ^2\,d\varOmega , \end{aligned}$$

where we denote the infinitesimal area element by $d\varOmega$ to avoid confusion with the abbreviation $x_k$ for the iterates in the first three sections of this paper. Alternatively, we will consider the output least squares type cost function term

56 $$\begin{aligned} J_{{\textit{mod}}}^{LS}(\sigma ,\mathbf {E},\mathbf {J})=\tfrac{1}{2}\int _\varOmega \left| \sigma \mathbf {E}-\mathbf {J}\right| ^2\,d\varOmega . \end{aligned}$$

Note that (56) is quadratic with respect to $\mathbf {J}$, thus quadratic with respect to $\psi$.

Excitation is imposed via the current *j* through the boundary, i.e., as Dirichlet boundary condition on $\psi$.

To incorporate the observations, we will consider the functionals

57 $$\begin{aligned} J_{obs}^{EIT}(\phi ;v)&= \tfrac{1}{2}\int _{\partial \varOmega } (\phi -v)^2\, d\varOmega \quad \text{ for } \text{ EIT, }\\ J_{obs_1}^{IAT}(\mathbf {E},\mathbf {J};\mathcal {H})&= \tfrac{1}{2}\int _\varOmega (\mathbf {J}\cdot \mathbf {E}-\mathcal {H})^2\, d\varOmega \quad \text{ or }\quad J_{obs_2}^{IAT}(\sigma ,\mathbf {E};\mathcal {H})\\&= \tfrac{1}{2}\int _\varOmega (\sigma |\mathbf {E}|^2-\mathcal {H})^2\, d\varOmega \quad \text{ for } \text{ IAT, }\\ J_{obs_1}^{GWF}(\phi ;p)&= \tfrac{1}{2}\Vert \phi -p\Vert _{H^s(\varOmega )}^2\quad \text{ or }\quad J_{obs_2}^{GWF}(\mathbf {E};g)\\&= \Vert \mathbf {E}-g\Vert _{L^2(\varOmega )}^2\quad \text{ for } \text{ GWF, } \end{aligned}$$

where again for GWF the use of the $H^s(\varOmega )$ norm or flux data can be justified by some pre-smoothing procedure applied to the given measurements.

Using these functionals as building blocks and incorporating the excitation via injection of the current *j* through the boundary we can write the above parameter identification problems in several minimization based formulations. We will now list a few of them, where *j* sometimes appears explicitly, sometimes in tangentially integrated form, meaning that for a parameterization $\varGamma$ of the boundary $\partial \varOmega$ (normalized to $\Vert \dot{\varGamma }\Vert =1$) we define $\alpha (\varGamma (s))=\int _0^s j(\varGamma (r))\, d r$ so that $\mathbf {J}\cdot \nu =\nabla ^\bot \psi \cdot \nu =\frac{d\alpha }{ds}=j$. Moreover we will sometimes work with smooth extensions $\phi _0$, $\psi _0$ of *v*, $\alpha$ to the interior of $\varOmega$. While, as already mentioned, the observation functional will depend on the application, we always have both $J_{mod}^{KV}$ and $J_{mod}^{LS}$ at our disposal to incorporate the model, thus will only write $J_{mod}$ below. There will also be versions based on an elimination of $\sigma$ by writing, for fixed $\phi ,\psi$, the minimizer of $J_{mod}$ with respect to $\sigma$ under the constraint $\underline{\sigma }\le \sigma \le \overline{\sigma }$ as

$$\begin{aligned} \sigma (\mathbf {E},\mathbf {J})=\max \left\{ \underline{\sigma },\min \left\{ \overline{\sigma },\tfrac{|\mathbf {J}|}{|\mathbf {E}|}\right\} \right\} \; \text{ pointwise } \text{ in }\;\varOmega . \end{aligned}$$

Alternatively to eliminating $\sigma$ it is also possible to eliminate $\phi ,\psi$ by writing them as $\phi (\sigma )$, $\psi (\sigma )$ minimizing $J_{mod}$ with respect to $\phi ,\psi$. This together with the integrated current $\alpha$ leads to boundary value problems for the elliptic PDE (54) and a similar PDE for $\psi$

$$\begin{aligned}&\phi (\sigma )\;\text{ solves } \left\{ \begin{array}{ll}\nabla \cdot (\sigma \nabla \phi )=0&{}\quad \text{ in }\;\varOmega \\ \phi =v&{}\quad \text{ on }\;\partial \varOmega \end{array}\right. \\&\phi _N(\sigma )\;\text{ solves } \left\{ \begin{array}{ll}\nabla \cdot (\sigma \nabla \phi )=0&{}\quad \text{ in }\;\varOmega \\ \nabla \phi \cdot \nu =j&{}\quad \text{ on }\;\partial \varOmega \, \quad \int _\varOmega \phi \, d\varOmega =0\end{array}\right. \\&\psi (\sigma )\;\text{ solves }\left\{ \begin{array}{ll}\nabla ^\bot \cdot \left( \frac{1}{\sigma }\nabla ^\bot \psi \right) =0&{}\quad \text{ in }\;\varOmega \\ \psi =\alpha &{}\quad \text{ on }\;\partial \varOmega \end{array}\right. \\&\mathbf {E}(\sigma ) = \tfrac{\nabla ^\bot \psi (\sigma )}{\sigma }\;\text{ pointwise } \text{ in }\;\varOmega \end{aligned}$$

(the latter two lines imply that $\nabla ^\bot \cdot \mathbf {E}(\sigma )=0$ so existence of $\phi$ such that $\mathbf {E}(\sigma )=\nabla \phi$) and corresponds to the classical reduced formulation of the inverse problem. Note that $\phi (\sigma )$ is only defined in case of *v* being observed, i.e., for EIT.

*EIT:*

$$\begin{aligned} (i)&\min _{\sigma ,\phi ,\psi }\left\{ J_{mod}(\sigma ,\nabla \phi ,\nabla ^\bot \psi )+\beta J_{obs}^{EIT}(\phi ;v) :\sigma \in L^2_{[\underline{\sigma },\overline{\sigma }]}(\varOmega ),\, \phi \in H_\diamondsuit ^1(\varOmega ), \psi \in H_0^1(\varOmega )+\psi _0\right\} \\ ({\textit{ii}})&\min _{\sigma ,\phi ,\psi }\left\{ J_{mod}(\sigma ,\nabla \phi ,\nabla ^\bot \psi ) : \sigma \in L^2_{[\underline{\sigma },\overline{\sigma }]}(\varOmega ),\, \phi \in H_0^1(\varOmega )+\phi _0,\, \psi \in H_0^1(\varOmega )+\psi _0\right\} \\ ({\textit{iii}})&\min _{\phi ,\psi }\{J_{mod}(\sigma (\nabla \phi ,\nabla ^\bot \psi ),\nabla \phi ,\nabla ^\bot \psi )+\beta J_{obs}^{EIT}(\phi ;v): \phi \in H_\diamondsuit ^1(\varOmega ), \psi \in H_0^1(\varOmega )+\psi _0\}\\ ({\textit{iv}})&\min _{\phi ,\psi }\{J_{mod}(\sigma (\nabla \phi ,\nabla ^\bot \psi ),\nabla \phi ,\nabla ^\bot \psi ) : \phi \in H_0^1(\varOmega )+\phi _0, \psi \in H_0^1(\varOmega )+\psi _0\}\\ (v)&\min _{\sigma }\left\{ J_{mod}(\sigma ,\nabla \phi (\sigma ),\nabla ^\bot \psi (\sigma )): \sigma \in L^2_{[\underline{\sigma },\overline{\sigma }]}(\varOmega )\right\} \\ ({\textit{vi}})&\min _{\sigma }\left\{ J_{obs}^{EIT}(\phi _N(\sigma );v):\sigma \in L^2_{[\underline{\sigma },\overline{\sigma }]}(\varOmega )\right\} \end{aligned}$$

*IAT:*

$$\begin{aligned}&(i)\;\min _{\sigma ,\phi ,\psi }\bigg \{J_{mod}(\sigma ,\nabla \phi ,\nabla ^\bot \psi )\\&\quad +\beta \left. \left\{ \begin{array}{l}J_{obs_1}^{IAT}(\nabla \phi ,\nabla ^\bot \psi ;\mathcal {H})\\ J_{obs_2}^{IAT}(\sigma ,\nabla \phi ;\mathcal {H})\end{array}\right. : \sigma \in L^2_{[\underline{\sigma },\overline{\sigma }]}(\varOmega ), \phi \in H_\diamondsuit ^1(\varOmega ), \psi \in H_0^1(\varOmega )+\psi _0\right\} \\&({\textit{ii}})\;\min _{\phi ,\psi }\bigg \{J_{mod}(\sigma (\nabla \phi ,\nabla ^\bot \psi ),\nabla \phi ,\nabla ^\bot \psi )\\&\quad +\beta \left. \left\{ \begin{array}{l}J_{obs_1}^{IAT}(\nabla \phi ,\nabla ^\bot \psi ;\mathcal {H})\\ J_{obs_2}^{IAT}(\sigma ,\nabla \phi ;\mathcal {H})\end{array}\right. : \phi \in H_\diamondsuit ^1(\varOmega ), \psi \in H_0^1(\varOmega )+\psi _0\right\} \\&({\textit{iii}})\;\min _{\sigma }\left\{ \left\{ \begin{array}{l}J_{obs_1}^{IAT}(\mathbf {E}(\sigma ),\nabla ^\bot \psi (\sigma );\mathcal {H})\\ J_{obs_2}^{IAT}(\sigma ,\mathbf {E}(\sigma );\mathcal {H})\end{array}\right. : \sigma \in L^2_{[\underline{\sigma },\overline{\sigma }]}(\varOmega )\right\} \end{aligned}$$

*GWF:*

$$\begin{aligned}&(i)\;\min _{\sigma ,\phi ,\psi }\bigg \{J_{mod}(\sigma ,\nabla \phi ,\nabla ^\bot \psi )\\&\quad +\beta \left. \left\{ \begin{array}{l}J_{obs_1}^{GWF}(\phi ;p)\\ J_{obs_2}^{GWF}(\nabla \phi ;g)\end{array}\right. : \sigma \in L^2_{[\underline{\sigma },\overline{\sigma }]}(\varOmega ), \, \phi \in H_\diamondsuit ^1(\varOmega ), \psi \in H_0^1(\varOmega )+\psi _0\right\} \\&({\textit{ii}})\;\min _{\phi ,\psi }\bigg \{J_{mod}(\sigma (\nabla \phi ,\nabla ^\bot \psi ),\nabla \phi ,\nabla ^\bot \psi )\\&\quad +\beta \left. \left\{ \begin{array}{l}J_{obs_1}^{GWF}(\phi ;p)\\ J_{obs_2}^{GWF}(\nabla \phi ;g)\end{array}\right. : \phi \in H_\diamondsuit ^1(\varOmega ), \psi \in H_0^1(\varOmega )+\psi _0\right\} \\&({\textit{iii}})\;\min _{\sigma }\left\{ J_{obs_2}^{GWF}(\mathbf {E}(\sigma );g): \sigma \in L^2_{[\underline{\sigma },\overline{\sigma }]}(\varOmega )\right\} \end{aligned}$$

where

$$\begin{aligned} L^2_{[\underline{\sigma },\overline{\sigma }]}(\varOmega )=\{\sigma \in L^2(\varOmega ) : \underline{\sigma }\le \sigma \le \overline{\sigma }\}, \quad H_\diamondsuit ^1(\varOmega ) =\left\{ \phi \in H^1(\varOmega ):\int _\varOmega \phi \, d\varOmega =0\right\} , \end{aligned}$$

and $\beta >0$ is a fixed parameter; we will simply set it to one in our computations. Note that $J_{mod}(\sigma ,\mathbf {E}(\sigma ),\nabla ^\bot \psi (\sigma ))=0$, therefore, the model term does not appear in the last instances of IAT and GWF, respectively. However, due to the bound constraints incorporated into the definition of $\sigma (\phi ,\psi )$, a nonzero value of $J_{mod}(\sigma (\phi ,\psi ),\nabla \phi ,\nabla ^\bot \psi )$ is possible, which is why it appears in the third and fourth instances of EIT. The sixth instance of EIT is just the classical reduced formulation.

As far as convexity is concerned, the Hessians of the functionals in (55), (56), (57) compute as

58 $$\begin{aligned}J_{mod}^{KV''}(\sigma ,\mathbf {E},\mathbf {J})[(h,\mathbf {v},\mathbf {w})^2]&=\int _\varOmega \left\{ \left| \tfrac{h}{\sqrt{\sigma }^3}\mathbf {J}+\sqrt{\sigma }\mathbf {v}-\tfrac{1}{\sqrt{\sigma }}\mathbf {w}\right| ^2\right. \\&\quad +2\tfrac{h}{\sigma }\left. \left( \sqrt{\sigma }\mathbf {E}-\tfrac{1}{\sqrt{\sigma }}\mathbf {J}\right) \cdot \mathbf {v}\right\} \,d\varOmega \\ J_{mod}^{LS''}(\sigma ,\mathbf {E},\mathbf {J})[(h,\mathbf {v},\mathbf {w})^2]&=\int _\varOmega \Bigl \{\left| h\mathbf {E}+\sigma \mathbf {v}-\mathbf {w}\right| ^2+h(\sigma \mathbf {E}-\mathbf {J})\cdot \mathbf {v}\Bigr \}\,d\varOmega \\ J_{obs}^{EIT''}(\phi ;v)[u^2] &= \int _{\partial \varOmega } u^2\, d\varOmega \\ J_{obs_1}^{IAT''}(\mathbf {E},\mathbf {J};\mathcal {H})[(\mathbf {v},\mathbf {w})^2] &= \int _\varOmega |\mathbf {w}\cdot \mathbf {E}+\mathbf {J}\cdot \mathbf {v}|^2 + (\mathbf {J}\cdot \mathbf {E}-\mathcal {H})\, \mathbf {w}\cdot \mathbf {v}\, d\varOmega \\ J_{obs_2}^{IAT''}(\sigma ,\mathbf {E};\mathcal {H})[(h,\mathbf {v})^2] &= \int _\varOmega (|h|\mathbf {E}+2\sigma \mathbf {E}\cdot \mathbf {v})^2\\&\quad +(\sigma |\mathbf {E}|^2-\mathcal {H})2(h\mathbf {v}\cdot \mathbf {E}+\sigma |v|^2)\, d\varOmega \\ J_{obs_1}^{GWF''}(\phi ;p)[u^2] &= \Vert u\Vert _{H^s(\varOmega )}^2\\ J_{obs_2}^{GWF''}(\mathbf {E};g)[\mathbf {v}^2] &= \Vert \mathbf {v}\Vert _{L^2(\varOmega )}^2. \end{aligned}$$

Thus, the Hessians of $J_{mod}^{KV}$, $J_{mod}^{LS}$, $J_{obs_1}^{IAT}$, $J_{obs_2}^{IAT}$ can only be guaranteed to be positive at their minimal points, whereas those of $J_{obs}^{EIT}$, $J_{obs_1}^{GWF}$, $J_{obs_2}^{GWF}$ are always positive. Since $J_{obs}^{EIT}$ only acts on the boundary, its additive combination with $J_{mod}^{KV}$ or $J_{mod}^{LS}$ cannot be expected to yield a globally convex functional. Likewise, combinations of $J_{obs_1}^{IAT}$ or $J_{obs_2}^{IAT}$ with $J_{mod}^{KV}$ or $J_{mod}^{LS}$ cannot be expected to be overall convex. This corresponds to the known fact that also for other formulations of EIT and IAT, the usual nonlinearity/convexity conditions fail to hold.

A combination satisfying the nonlinearity assumption (49) and therefore also (47), (18) is GWF with

$$\begin{aligned} J^\delta (\sigma ,\phi ,\psi )=J_{mod}^{LS}(\sigma ,\nabla \phi ,\nabla ^\bot \psi )+\beta J_{obs}^{GWF}(\nabla \phi ;g^\delta ). \end{aligned}$$

To verify this, we show that (29), (50) is satisfied for $$F(\sigma ,\phi ,\psi )=\left( \begin{array}{c}\sigma \nabla \phi -\nabla ^\bot \psi \\ \nabla \phi \end{array}\right)$$ by estimating (with the abbreviations $\mathbf {E}=\nabla \psi$, $\mathbf {J}=\nabla ^\bot \psi$)

$$\begin{aligned}&|\langle F(x_+)-F(x)-F'(x)(x_+-x),F(x)-y^\delta \rangle | \\&\quad =\int _\varOmega (\sigma _+-\sigma )(\mathbf {E}_+-\mathbf {E})(\sigma \mathbf {E}-\mathbf {J})\, d\varOmega \\&\quad \le \Vert \sigma _+-\sigma \Vert _{L^\infty (\varOmega )} \Vert \mathbf {E}_+-\mathbf {E}\Vert _{L^2(\varOmega )} \Vert \sigma \mathbf {E}-\mathbf {J}\Vert _{L^2(\varOmega )}\\&\quad \le (\overline{\sigma }-\underline{\sigma }) \sqrt{\Vert \sigma _+\mathbf {E}_+-J_+-\sigma \mathbf {E}+J\Vert _{L^2(\varOmega )}^2+\Vert \mathbf {E}_+-\mathbf {E}\Vert _{L^2(\varOmega )}^2} \\&\qquad \cdot \sqrt{\Vert \sigma \mathbf {E}-\mathbf {J}\Vert _{L^2(\varOmega )}^2+\Vert \mathbf {E}-g^\delta \Vert _{L^2(\varOmega )}^2}\\&\quad = (\overline{\sigma }-\underline{\sigma }) \Vert F(x_+)-F(x)\Vert \, \Vert F(x)-y^\delta \Vert \\ \end{aligned}$$

which directly implies (50) with $c_{tc}=\frac{\overline{\sigma }-\underline{\sigma }}{\sup _{x\in \tilde{M}^\delta }\Vert F'(x)\Vert }$ and hence (29) with $\gamma = 1-c_{tc}-\kappa$ provided $(1+c_{tc})\Vert F(x)-y^\delta \Vert \le 2\sqrt{\kappa \eta (\delta )}$. In order to obtain a finite value of

$$\begin{aligned} \sup _{x\in \tilde{M}^\delta }\Vert F'(x)\Vert =\sup _{(\sigma ,\phi ,\psi )\in \tilde{M}^\delta }\sup _{(h,\mathbf {v},\mathbf {w})\in L^2(\varOmega )^5\setminus \{0\}}\frac{\int _\varOmega (h\nabla \phi +\sigma \mathbf {v}-\mathbf {w})\cdot (\sigma \nabla \phi -\nabla ^\bot \psi )\, d\varOmega }{\Vert (h,\mathbf {v},\mathbf {w})\Vert _{L^2(\varOmega )^5}} \end{aligned}$$

we choose $\tilde{M}^\delta$ to be a bounded subset of $L^\infty (\varOmega )\times W^{1,\infty }(\varOmega )\times H^1(\varOmega )$ with an a priori bound satisfied by the exact solution of the inverse problem.

## Numerical results for IAT, GWF, and EIT

In this section, we will provide some numerical results for the problem of identifying the conductivity $\sigma$ in (54). As already mentioned, we will work with the more realistic complete electrode model (CEM) instead of idealized continuous boundary excitation and observations. Moreover, we will focus on the hybrid tomographic application IAT and we will only show one set of reconstructions of GWF and EIT. More extensive numerical tests for IAT but also for GWF and EIT can be found in the PhD thesis [12].

### The complete electrode model and setting for the cost functions

**Fig. 1:**
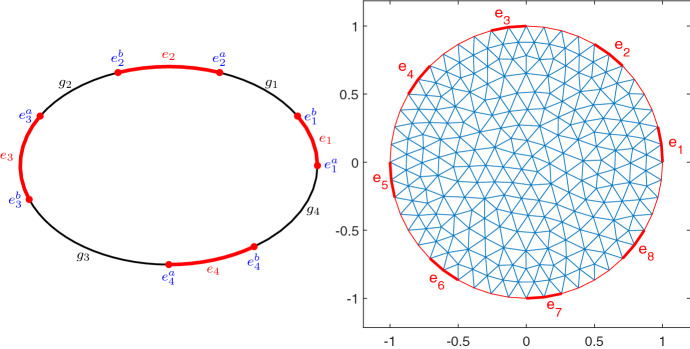
Left: Electrodes (in boldface red) on the boundary with $L=4$; right: finite element discretization with 8 electrodes (Color figure online)

In the complete electrode model (CEM) current is fed in through a finite number of electrodes, $e_1, \dots , e_L$, see Fig. 1. In case of boundary measurements, as relevant for EIT, they are also taken at these electrodes.

Let $J_i$, $E_i$, $i = 1,2, \dots , I$, be the current density and the electric field in the *i*th measurement, and let $\phi _i$, $\psi _i$ be the potentials for $J_i$, $E_i$; then they must satisfy

59a $$\begin{aligned} \sqrt{\sigma }\nabla \phi _i - \frac{1}{\sqrt{\sigma }} \nabla ^\bot \psi _i = 0&\quad \text {in}\; \varOmega , \end{aligned}$$

59b $$\begin{aligned} \phi _i + z_\ell \nabla ^\bot \psi _i \cdot \nu = v_{\ell ,i}&\quad \text {on}\; e_\ell , \ell =1,2,\dots ,L, \end{aligned}$$

59c $$\begin{aligned} \int _{e_\ell } \nabla ^\bot \psi _i \cdot \nu {~\mathrm {d}}s = j_{\ell ,i}&\quad \text {for}\; \ell =1,2,\dots ,L, \end{aligned}$$

59d $$\begin{aligned} \nabla ^\bot \psi _i \cdot \nu = 0&\quad \text {on}\; \partial \varOmega \backslash \cup _{\ell =1}^L e_\ell , \end{aligned}$$

$$\forall i= 1, 2, \dots , I$$, where

- $j_{\ell ,i}$, $v_{\ell ,i}$ are the applied current and measured voltage on $e_\ell$ at the *i*th measurement,
- $\{z_\ell \}_{\ell =1}^{L}$ is the set of positive contact impedances.

By assuming $\psi (e_i^a) = 0$ and using (59c), (59d) (59b), we get

$$\begin{aligned} \begin{array}{ll} \psi _i|_{g_{\ell }} = {\bar{j}}_{\ell ,i}, &{}\quad \forall \ell \in \{1,\dots ,L\}, \\ \displaystyle \int _{e_\ell ^a}^x \phi _i {~\mathrm {d}}s - z_\ell \psi _i(x) = \bar{v}_{\ell ,i}(x), &{}\quad \forall x \in e_\ell , \forall \ell \in \{1,\dots ,L\}, \end{array} \end{aligned}$$

where ${\bar{j}}_{\ell ,i} = -\sum _{k=1}^{\ell } j_{k,i}$, $\bar{v}_{\ell ,i}(x) = -z_\ell \Big ( -\sum _{k=1}^{\ell -1} j_{k,i} \Big )+ v_{\ell ,i} d_{e_\ell } (x)$ and $d_{e_\ell } (x)$ is the length of $e_\ell$ from $e_\ell ^a$ to *x*.

In the case of EIT, the data $(j_{\ell ,i}, v_{\ell ,i})_{\ell ,i} \in \mathbb {R}^{2LI}$ can be considered as $({\bar{j}}_{\ell ,i}, \bar{v}_{\ell ,i})_{\ell ,i} \in \prod _{i=1}^I \prod _{\ell =1}^L (L^2(g_\ell ) \times L^2(e_\ell ))$ and the cost function part corresponding to observations is chosen as $J^{EIT}_{obs}: H^1(\varOmega )^{2I} \rightarrow \mathbb {R}$,

60 $$\begin{aligned} J^{EIT}_{obs}(\varPhi , \varPsi ; {\bar{j}}, {\bar{v}})&= \frac{1}{2} \sum _{i=1}^I \sum _{\ell =1}^L \left( \int _{g_\ell } |\psi _i - {\bar{j}}_{\ell ,i}|^2 {~\mathrm {d}}s\right. \\&\quad + \left. \int _{e_\ell } \left| \int \phi _i|_{e_\ell } - z_{\ell } \psi _i - {\bar{v}}_{\ell ,i} \right| ^2 {~\mathrm {d}}s \right) , \end{aligned}$$

where $$\varPhi = (\phi _i)_i$$, $$\varPsi = (\psi _i)_i$$, $${\bar{j}} = (\bar{j}_{\ell ,i})_{\ell ,i}$$, $${\bar{v}} = ({\bar{v}}_{\ell ,i})_{\ell ,i}$$ and $$\int \phi _i|_{e_\ell }: e_\ell \rightarrow \mathbb {R}, x \mapsto \int _{e_\ell ^a}^x \phi _i {~\mathrm {d}}s$$.

In the cases of IAT and GWF, instead of $(j_{\ell ,i}, v_{\ell ,i})_{\ell ,i}$, we observe $\mathcal {H} = (\mathcal {H}_i)_i = (\sigma |\nabla \phi _i|^2)_i$ or $p= (p_i)_i = (\phi _i)_i$, respectively and the cost function part corresponding to these observations is $J^{IAT}_{obs} {/J^{GWF}_{obs}} : L^2(\varOmega ) \times H^1(\varOmega )^I \rightarrow \mathbb {R}$,

61 $$\begin{aligned} J^{IAT}_{obs}(\sigma , \varPhi ; \mathcal {H})&= \frac{1}{2} \sum _{i=1}^I \int _\varOmega \Big | \sigma |\nabla \phi _i|^2 - \mathcal {H}_i \Big |^2 {~\mathrm {d}}\varOmega ,\\ J^{GWF}_{obs_1}(\sigma , \varPhi ; p)&= \frac{1}{2} \sum _{i=1}^I \int _\varOmega \big | \phi _i - p_i \big |^2 {~\mathrm {d}}\varOmega . \end{aligned}$$

In all three cases EIT, IAT, GWF, we choose the cost function part corresponding to the model as $J^{KV}_{mod}: L^2(\varOmega ) \times H^1(\varOmega )^{2I} \rightarrow \mathbb {R}$,

62 $$\begin{aligned} J^{KV}_{mod}(\sigma , \varPhi , \varPsi ) = \frac{1}{2} \sum _{i=1}^I \int _\varOmega \left| \sqrt{\sigma } \nabla \phi _i - \frac{1}{\sqrt{\sigma }} \nabla ^\bot \psi _i \right| ^2 {~\mathrm {d}}\varOmega \end{aligned}$$

and combine it with the observation part to

63 $$\begin{aligned} J^{IAT}(\sigma , \varPhi , \varPsi ) = J^{KV}_{mod}(\sigma , \varPhi , \varPsi ) + J^{IAT}_{obs}(\sigma , \varPhi ; \mathcal {H}) \end{aligned}$$

64 $$\begin{aligned} J^{GWF}(\sigma , \varPhi , \varPsi ) = J^{KV}_{mod}(\sigma , \varPhi , \varPsi ) + J^{GWF}_{obs_1}(\varPhi ;p) \end{aligned}$$

65 $$\begin{aligned} J^{EIT}(\sigma , \varPhi , \varPsi ) = J^{KV}_{mod}(\sigma , \varPhi , \varPsi ) + J^{EIT}_{obs}(\varPhi , \varPsi ; {\bar{j}}, {\bar{v}}) \end{aligned}$$

on the admissible sets

$$\begin{aligned} M^{IAT}_{{\mathrm {ad}}} {= M^{GWF}_{{\mathrm {ad}}}} = M^{EIT}_{{\mathrm {ad}}} = L^2_{[\underline{\sigma }, \overline{\sigma }]} (\varOmega ) \times H_\diamondsuit ^1(\varOmega ) \times H_0^1(\varOmega )+\varPhi _0. \end{aligned}$$

As in the previous section, besides the resulting all-at-once versions [cf. EIT (i), (ii) and IAT (i)] we also consider some of the reduced versions of the cost function. [For GWF we will only show results with the all-at-once version (i)]

The first version involves eliminating $\sigma$ from the cost function [cf. EIT (iii), (iv) and IAT (ii)], by defining, for given $\varPhi ,\varPsi$, the corresponding $\sigma$ by

66 $$\begin{aligned} \sigma (\varPhi ,\varPsi )&= {{\,\mathrm{argmin}\,}}_{\sigma } \left\{ \frac{1}{2} \sum _{i=1}^{I} \int _\varOmega \left| \sqrt{\sigma } \nabla \phi _i - \frac{1}{\sqrt{\sigma }} \nabla ^\bot \psi _i \right| ^2 {~\mathrm {d}}\varOmega : \sigma \in L^2_{[\underline{\sigma }, \overline{\sigma }]} (\varOmega ) \right\} \\&= {{\,\mathrm{argmin}\,}}_{\sigma } \left\{ \sum _{i=1}^{I} \int _\varOmega \left( \sigma |\nabla \phi _i|^2 + \frac{1}{\sigma } |\nabla ^\bot \psi _i|^2 \right) {~\mathrm {d}}\varOmega : \sigma \in L^2_{[\underline{\sigma }, \overline{\sigma }]} (\varOmega ) \right\} \end{aligned}$$

or explicitly

$$\begin{aligned} \sigma (\varPhi ,\varPsi ) =\min \left\{ \overline{\sigma }, \max \left\{ \underline{\sigma }, \sqrt{\frac{\sum _{i=1}^I |\nabla ^\bot \psi _i|^2}{\sum _{i=1}^I |\nabla \phi _i|^2}} \right\} \right\} . \end{aligned}$$

For the case of IAT, we set

67 $$\begin{aligned} J^{IAT}_\sigma (\varPhi , \varPsi ; \mathcal {H}) = J^{KV}_{mod} (\sigma (\varPhi , \varPsi ), \varPhi , \varPsi ) + \beta J^{IAT}_{obs} (\sigma (\varPhi , \varPsi ), \varPhi ; H) \end{aligned}$$

for the case of EIT,

68 $$\begin{aligned} J^{EIT}_\sigma (\varPhi , \varPsi ; {\bar{j}}, {\bar{v}}) = J^{KV}_{mod} (\sigma (\varPhi , \varPsi ), \varPhi , \varPsi ) + \beta J^{EIT}_{obs} (\varPhi , \varPsi ; {\bar{j}}, {\bar{v}}) \end{aligned}$$

and in all cases

$$\begin{aligned} M^{IAT}_{\sigma ,{\mathrm {ad}}} = M^{EIT}_{\sigma ,{\mathrm {ad}}} = H_\diamondsuit ^1(\varOmega ) \times H_0^1(\varOmega )+\varPhi _0. \end{aligned}$$

Note that in spite of the minimizing pre-definition of $\sigma (\varPhi , \varPsi )$, the model cost function part may be nonzero due to the constraints and therefore still needs to be taken into account.

The second alternative cost function involves eliminating $(\varPhi , \varPsi )$ from the cost function [cf. EIT (v), (vi) and IAT (iii)] by means of the weak form of the CEM PDE (59)

69 $$\begin{aligned}&\int _\varOmega \sigma \nabla \phi _i \cdot \nabla p {~\mathrm {d}}\varOmega + \sum _{\ell =1}^L \frac{1}{z_\ell } \int _{e_\ell } (\phi _i - v_{\ell ,i}) (p - \xi _{\ell }) {~\mathrm {d}}s \\&\quad = \sum _{\ell =1}^L j_{\ell ,i} \xi _\ell , \forall (p,\xi ) \in H^1 (\varOmega ) \times \mathbb {R}^L, \end{aligned}$$

and $\nabla ^\bot \psi _i = \sigma \nabla \phi _i, \forall i \in \{1,\dots ,I\}$, which leads to $J^{KV}_{mod} (\sigma , \varPhi (\sigma ), \varPsi (\sigma )) = 0$. Hence, we have, for the case of IAT,

70 $$\begin{aligned} J^{IAT}_{(\varPhi ,\varPsi )}(\sigma ; \mathcal {H}) = J^{IAT}_{obs} (\sigma , \varPhi (\sigma ); \mathcal {H}) = \frac{1}{2} \sum _{i=1}^I \int _\varOmega \Big ( \sigma |\nabla \phi _i(\sigma )|^2 - \mathcal {H}_i \Big )^2 {~\mathrm {d}}\varOmega ; \end{aligned}$$

for the case of EIT,

71 $$\begin{aligned} J^{EIT}_{(\varPhi ,\varPsi )}(\sigma ; v) = \frac{1}{2} \sum _{i=1}^I \sum _{\ell =1}^L \big | v_{\ell ,i}(\sigma ) - v_{\ell ,i} \big |^2, \end{aligned}$$

where $v(\sigma ) = (v_{\ell ,i}(\sigma ))_{i \in \{1,\dots ,I\},\ell \in \{1,\dots ,L\}}$ is the solution to (69) and

$$\begin{aligned} M^{IAT}_{(\varPhi , \varPsi ),{\mathrm {ad}}} = M^{EIT}_{(\varPhi , \varPsi ),{\mathrm {ad}}} = L^2_{[\underline{\sigma }, \overline{\sigma }]} (\varOmega ) \end{aligned}$$

Summarizing, our tests comprise three different formulations: all-at-once, eliminate $\sigma$, eliminate $(\varPhi , \varPsi )$. The latter corresponds to the conventional reduced approach, whereas the former two are new.

### Implementation using the finite element method in Matlab

In order to generate synthetic data by solving the CEM PDE (59) we use the finite element method. In all our computations, $\varOmega$ is the unit circle in $\mathbb {R}^2$ with eight identical electrodes ($L=8$) denoted by $e_1, \dots , e_8$ attached equidistantly on its boundary (see Fig. 1). The domain $\varOmega$ is discretized by a regular finite element mesh defined by nodes $P_k, k\in \{1,\dots , N_{\mathrm {node}}= 913\}$ and elements $\varOmega _h, h \in \{1,\dots ,N_{\mathrm {element}}=432\}$. The ansatz spaces $L^2 (\varOmega )$ for $\sigma$ and $H^1 (\varOmega )$ for $\phi ,\psi$ are approximated by piecewise constant and continuous piecewise quadratic finite element spaces ${\tilde{L}}^2 (\varOmega )$ and ${\tilde{H}}^1 (\varOmega )$, respectively.

With $L=8$ electrodes, there are $N_{\mathrm {meas}}= 28$ possible combinations of excitations—we will use some of them to reconstruct $\sigma$ later. At the *i*th measurement, we impose the injected current $(j_{\ell ,i})_{\ell =1}^L$ with

$$\begin{aligned} \left\{ \begin{array}{ll} j_{\ell _1,i} = 1, j_{\ell _2,i} = -1, &{}\quad \text {if}\;\ell _1<\ell _2\;\hbox {and}\;\{\ell _1,\ell _2\}\;\hbox {is the}\;i\hbox {th element of}\\ &{}\quad \text {the family of 2-elements subsets of}\; \{1,\dots ,8\}, \\ j_{\ell ,i} = 0, &{}\quad \text {otherwise,} \end{array} \right. \end{aligned}$$

at the electrodes and then solve the Galerkin discretized weak form (cf. (69))

72 $$\begin{aligned}&\int _\varOmega \sigma ^{\mathrm {ex}}\nabla \phi _i \cdot \nabla p {~\mathrm {d}}x + \sum _{\ell =1}^L \frac{1}{z_\ell } \int _{e_\ell } (\phi _i - v_{\ell ,i}) (p - \xi _{\ell }) {~\mathrm {d}}s = \sum _{\ell =1}^L j_{\ell ,i} \xi _\ell , \\&\quad \forall (p,\xi ) \in {\tilde{H}}^1 (\varOmega ) \times \mathbb {R}^L, \forall i \in \{1,\dots ,N_{\mathrm {meas}}\} \end{aligned}$$

to find $(\phi ^{\mathrm {ex}}_i,(v^{\mathrm {ex}}_{\ell ,i}))$ and the corresponding exact data

$$\begin{aligned} \mathcal {H}^{\mathrm {ex}}= (\sigma ^{\mathrm {ex}}|\nabla \phi ^{\mathrm {ex}}_1|^2, \dots , \sigma ^{\mathrm {ex}}|\nabla \phi ^{\mathrm {ex}}_{N_{\mathrm {meas}}}|^2)\;\text{ for } \text{ IAT; } \quad (j_{\ell ,i}, v^{\mathrm {ex}}_{\ell ,i})_{\ell ,i}\;\text{ for } \text{ EIT. } \end{aligned}$$

The synthetic measured data is generated by adding random noise such that

$$\begin{aligned} |\mathcal {H}^\delta _i - \mathcal {H}^{\mathrm {ex}}_i| \le \delta |\mathcal {H}^{\mathrm {ex}}_i|, \quad \forall i\;\text{ for } \text{ IAT; } \quad |v^\delta _{\ell ,i} - v^{\mathrm {ex}}_{\ell ,i}| \le \delta |v^{ex}_{\ell ,i}| \quad \text{ for } \text{ EIT, } \end{aligned}$$

which in an obvious way defines the noisy versions $J^\delta$, $J_\sigma ^\delta$, $J_{\varPhi ,\varPsi }^\delta$ and $M^\delta$, $M_{\sigma ,{\mathrm {ad}}}^\delta$, $M_{(\varPhi , \varPsi ),{\mathrm {ad}}}^\delta$ of the cost functions and admissible sets, respectively. In our tests we consider three values of $\delta$: $\delta =0$, $\delta =0.01$ and $\delta =0.1$.

To avoid an inverse crime, we used a coarser mesh in our reconstructions.

In (69) we set the value of contact impedances $z_\ell$, $\ell \in \{1,\dots ,L\}$, to 0.1.

The test case considered in all of our computational results is defined by a constant inclusion on a constant background

$$\begin{aligned} \sigma ^{{\mathrm {ex}}} (x) = \left\{ \begin{array}{ll} 5, &{}\;\text {in}\; \varOmega _h\;\text {if}\;\varOmega _h \subset B_{0.5}(-0.3,-0.1)\\ 2, &{}\;\text {otherwise,} \end{array}\right. \end{aligned}$$

where $B_r(p_1,p_2) \subset \mathbb {R}^2$ is the ball centered at $(p_1,p_2)$ with radius *r*. For IAT we also show results with two inclusions

73 $$\begin{aligned} \sigma ^{{\mathrm {ex}}_{2incl}} (x) = \left\{ \begin{array}{ll} 5, &{}\; \text {in}\; \varOmega _h\;\text {if}\; \varOmega _h \subset B_{0.5}(-0.3,-0.1)\\ 4.25, &{}\; \text {in}\; \varOmega _h\; \text {if}\; \varOmega _h \subset B_{0.3}(0.4,0.5)\\ 2, &{}\; \text {otherwise,} \end{array}\right. \end{aligned}$$

With each of the three above mentioned cost function combinations (all-at-once, eliminated $\sigma$, eliminated $(\varPhi ,\varPsi )$, the iterates $x_k=(\sigma _k,\varPhi _k,\varPsi _k)$, or $x_k=(\varPhi _k,\varPsi _k)$, or $x_k=\sigma _k$, are defined by the projected gradient method (12) from Sect. 2 where $\mu _k$ is found by an Armijo back tracking line search. Details on computation of the gradients of the various cost functions can be found in [12]. The iteration is stopped by the discrepancy principle (20) in the noisy case and as soon as the step size fell below a value $\epsilon _\mu$ (which we set to $10^{10}$ in our tests) in case of exact data.

### Numerical results for IAT

We consider four cases of excitations, namely

- $I=1$, with $j_{1,1}=1$, $j_{5,1}=-1$ and $j_{k,1}=0$ otherwise;
- $I=2$, with $j_{1,1}=j_{3,2}=1$, $j_{5,1}=j_{7,2}=-1$, and $j_{k,i}=0$ otherwise;
- $I=4$, with $j_{1,1}=j_{3,2}=j_{2,3}=j_{4,4}=1$, $j_{5,1}=j_{7,2}=j_{6,3}=j_{8,4}=-1$ and $j_{k,i}=0$ otherwise.
- $I=28$, with all $$(\begin{array}{c} 8\\ 2 \end{array})$$ combinations of setting $j_{k,i}=1$, $j_{\ell ,i}=-1$ for $k\not =\ell \in \{1,\ldots ,8\}$

The starting value is set to the mean value of the maximal and minimal value for the conductivity $\sigma _0 = \frac{1}{2} (\underline{\sigma } + \overline{\sigma })$ and $\varPhi _0, \varPsi _0$, if necessary, are gained from the weak form (69) where $\sigma$ is replaced by $\sigma _0$.

Tables 1, 2 and 3 show the data about *the number of iterations*, *the error *$\Vert \sigma _{end} - \sigma ^{{\mathrm {ex}}} \Vert _{L^2 (\varOmega )}$, *the CPU time* (in seconds) and *the CPU time for each iteration* for various versions of cost functions.

**Fig. 2:**
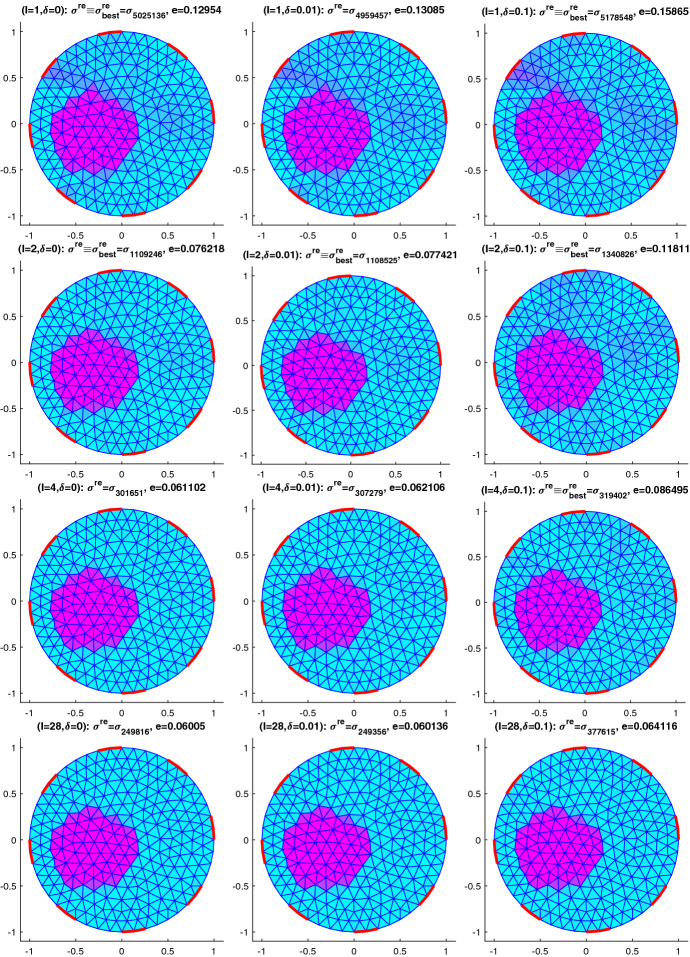
Reconstructions of $\sigma$ from all-at-once version of cost function IAT (62), (61), in cases $I=1$, $I=2$, $I=4$, $I=28$ (top to bottom) for $\delta =0$, $\delta =0.01$, $\delta =0.1$ (left to right)

**Fig. 3:**
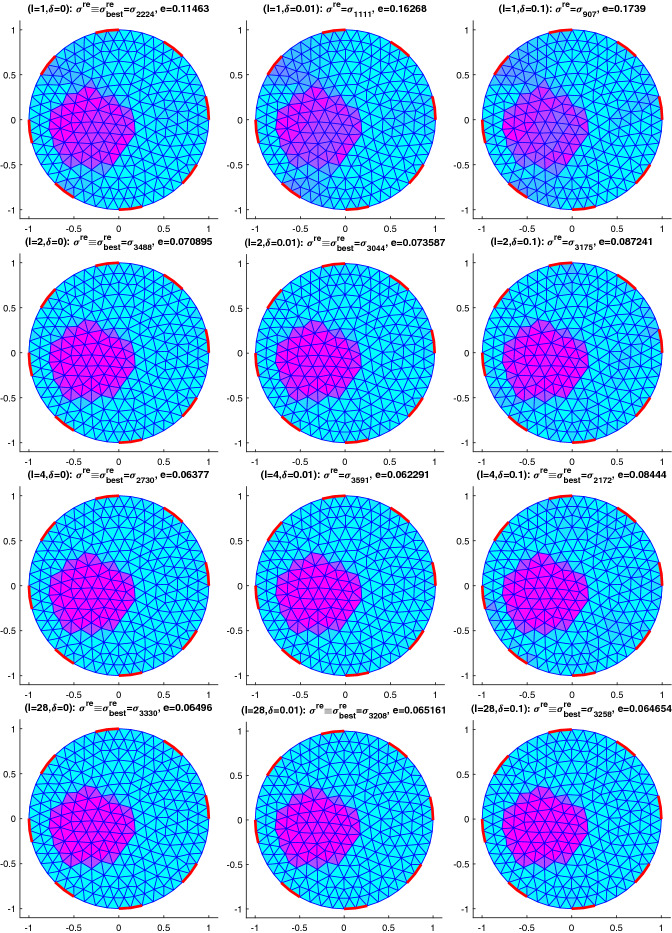
Reconstructions of $\sigma$ from eliminating-$\sigma$ version of cost function IAT (67), in cases $I=1$, $I=2$, $I=4$, $I=28$ (top to bottom) for $\delta =0$, $\delta =0.01$, $\delta =0.1$ (left to right)

**Fig. 4:**
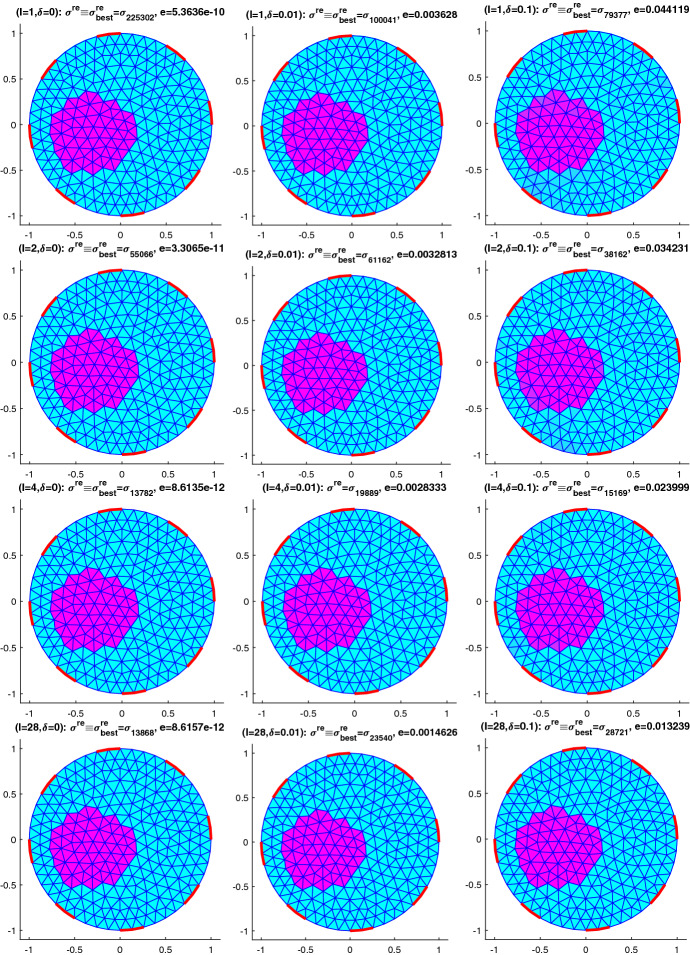
Reconstructions of $\sigma$ from eliminating-$\varPhi -\varPsi$ version of cost function IAT (70), in cases $I=1$, $I=2$, $I=4$, $I=28$ (top to bottom) for $\delta =0$, $\delta =0.01$, $\delta =0.1$ (left to right)

In Figs. 2, 3 and 4, we display some pictures of reconstructions.

Evidently, reconstructions improve with more information—that is, larger *I*—and better quality data—that is, lower $\delta$. Also, the computationally more expensive “eliminate $(\varPhi ,\varPsi )$” version that requires solution of elliptic boundary value problems provides better results than the cheaper “eliminate $\sigma$” or all-at-once versions. This is clearly visible in the relative errors (column 3 of Tables 1, 2 and 3) and CPU times (last two columns of Tables 1, 2 and 3). Surprisingly, in spite of the fact that IAT cannot be shown to satisfy the assumption (49) due to nonlinearity of the observation operator, results are better for IAT than for GWF, see Fig. 8, to be compared with Fig. 2

**Fig. 5:**
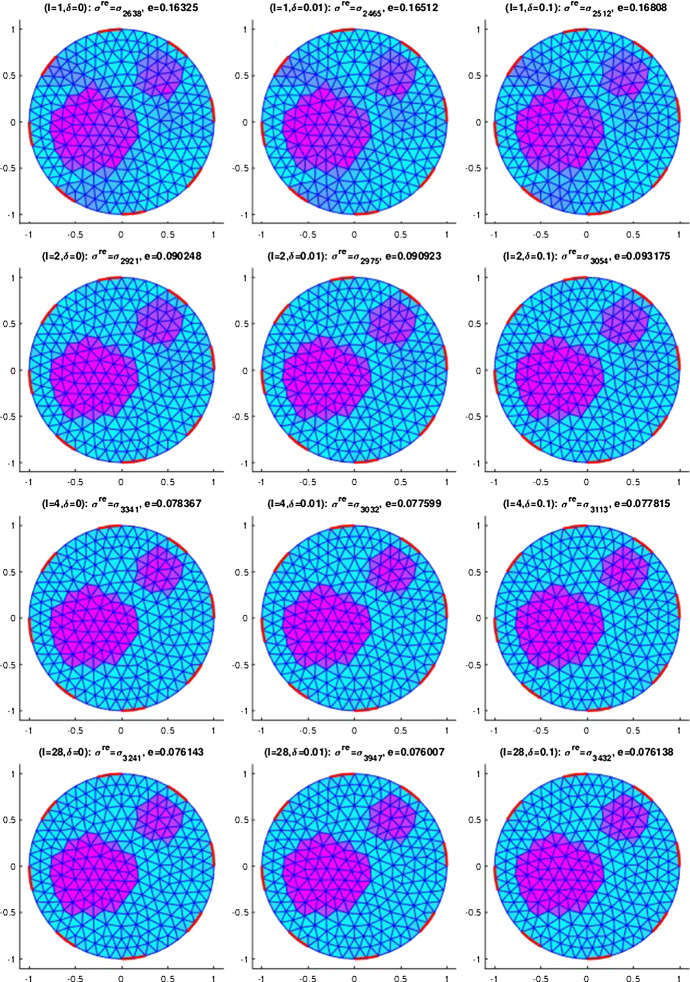
Reconstructions of $\sigma$ from all-at-once version of cost function IAT (62), (61), in cases $I=1$, $I=2$, $I=4$, $I=28$ (top to bottom) for $\delta =0$, $\delta =0.01$, $\delta =0.1$ (left to right) for the two inclusions example (73)

**Fig. 6:**
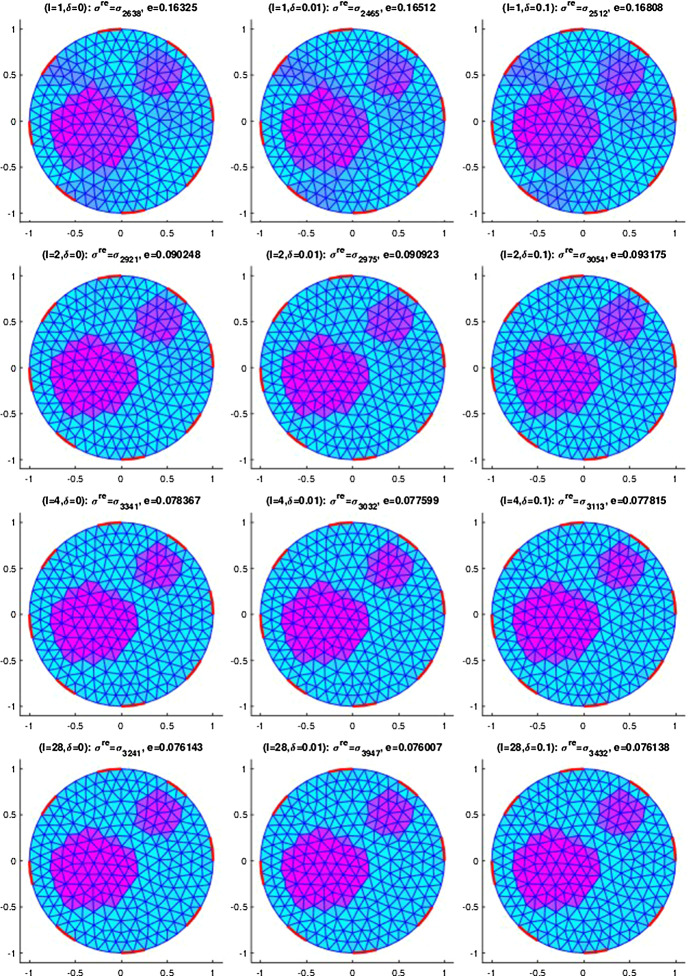
Reconstructions of $\sigma$ from eliminating-$\sigma$ version of cost function IAT (67), in cases $I=1$, $I=2$, $I=4$, $I=28$ (top to bottom) for $\delta =0$, $\delta =0.01$, $\delta =0.1$ (left to right) for the two inclusions example (73)

**Fig. 7:**
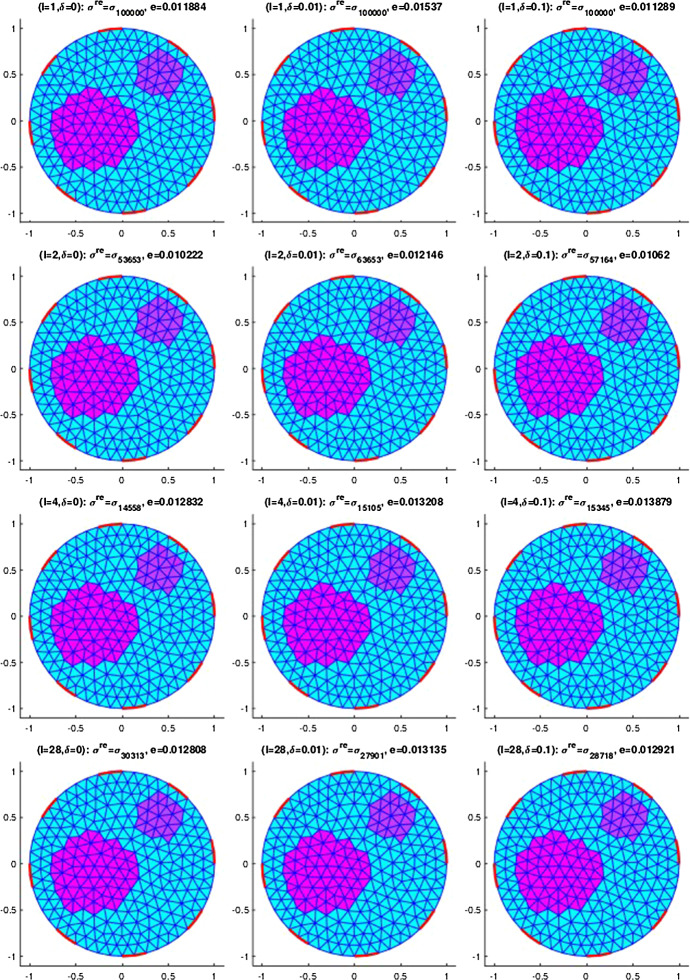
Reconstructions of $\sigma$ from eliminating-$\varPhi -\varPsi$ version of cost function IAT (70), in cases $I=1$, $I=2$, $I=4$, $I=28$ (top to bottom) for $\delta =0$, $\delta =0.01$, $\delta =0.1$ (left to right) for the two inclusions example (73)

Finally, we also show some reconstructions of two inclusions in Figs. 5, 6 and 7.

**Table 1:** IAT, all-at-once version (62), (61)

All-at-once version IAT		Number of iterations	$L^2$ error $\Vert \sigma _{end} - \sigma ^{{\mathrm {ex}}}\Vert$	CPU-time (in seconds)	CPU-time per iteration
$I=1$	$\delta=0$	5,025,130	0.65872	943,527	0.18776
	$\delta=0.01$	4,959,452	0.66537	930,984	0.18772
	$\delta=0.1$	5,178,542	0.80676	984,851	0.19018
$I=2$	$\delta=0$	1,109,245	0.38757	266,270	0.24005
	$\delta=0.01$	1,114,829	0.38746	264,697	0.23743
	$\delta=0.1$	1,520,239	0.56700	344,894	0.22687
$I=4$	$\delta=0$	301,651	0.31070	73,664	0.24420
	$\delta=0.01$	308,170	0.31496	74,474	0.24166
	$\delta=0.1$	326,561	0.41261	79,693	0.24404
$I=28$	$\delta=0$	249,816	0.30535	82,681	0.33097
	$\delta=0.01$	245,306	0.30675	81,263	0.33127
	$\delta=0.1$	292,914	0.32676	96,378	0.32903

**Table 2:** IAT, eliminating-$\sigma$ version (67)

Eliminating-$\sigma$ version, IAT		Number of iterations	$L^2$ error $\Vert \sigma _{end} - \sigma ^{{\mathrm {ex}}}\Vert$	CPU-time (in seconds)	CPU-time per iteration
$I=1$	$\delta=0$	2224	0.58289	583	0.26228
	$\delta=0.01$	1230	0.79777	319	0.25946
	$\delta=0.1$	840	0.94467	215	0.25636
$I=2$	$\delta=0$	3488	0.36050	1232	0.35321
	$\delta=0.01$	2894	0.37353	920	0.31786
	$\delta=0.1$	2645	0.45520	959	0.36274
$I=4$	$\delta=0$	2730	0.32427	801	0.29349
	$\delta=0.01$	3393	0.32152	1254	0.36959
	$\delta=0.1$	1836	0.43352	562	0.30589
$I=28$	$\delta=0$	3330	0.33032	1451	0.43561
	$\delta=0.01$	3251	0.33105	1427	0.43900
	$\delta=0.1$	3300	0.34439	1511	0.45787

**Table 3:** IAT, eliminating-$(\varPhi ,\varPsi )$ version (70)

Eliminating-$(\varPhi ,\varPsi )$ version, IAT		Number of iterations	$L^2$ error $\Vert \sigma _{end} - \sigma ^{{\mathrm {ex}}}\Vert$	CPU-time (in seconds)	CPU-time per iteration
$I=1$	$\delta=0$	225,302	2.73e−09	147,936	0.65661
	$\delta=0.01$	100,041	0.01845	83,302	0.83268
	$\delta=0.1$	79,377	0.22434	38,043	0.47927
$I=2$	$\delta=0$	55,066	1.68e−10	30,161	0.54772
	$\delta=0.01$	61,162	0.01669	61,202	1.00065
	$\delta=0.1$	38,162	0.17407	41,314	1.08260
$I=4$	$\delta=0$	13,782	4.38e−11	10,469	0.75958
	$\delta=0.01$	19,889	0.01441	27,064	1.36076
	$\delta=0.1$	15,169	0.12203	19,721	1.30006
$I=28$	$\delta=0$	13,868	4.38e−11	49,005	3.53365
	$\delta=0.01$	23,540	0.00744	96,096	4.08226
	$\delta=0.1$	28,721	0.06732	120,978	4.21217

### Numerical results for GWF

**Fig. 8:**
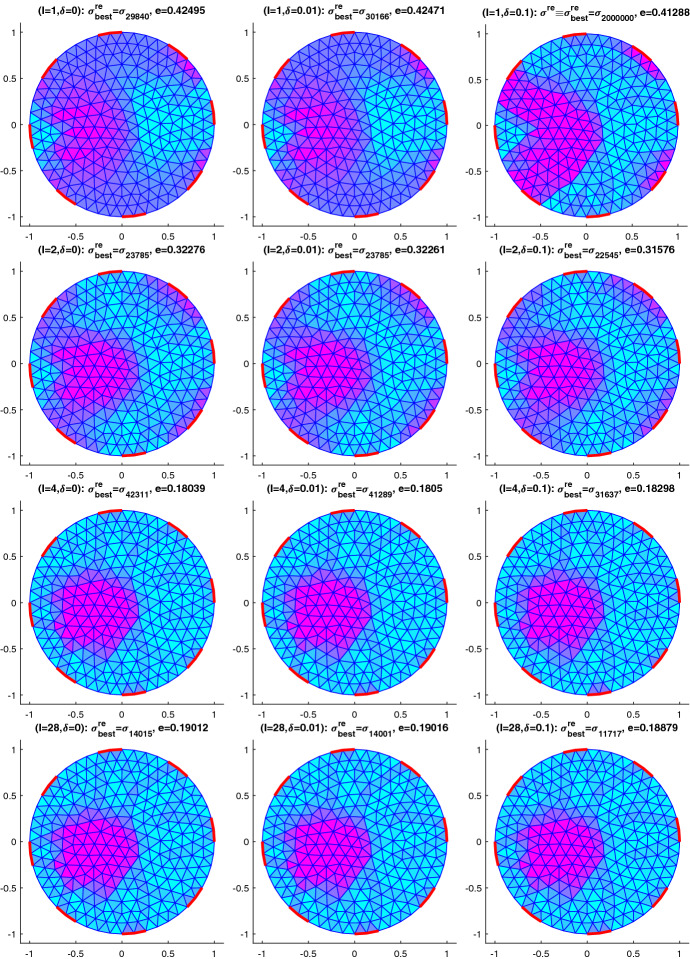
Reconstructions of $\sigma$ from all-at-once version of cost function GWF (62), (61), in cases $I=1$, $I=2$, $I=4$, $I=28$ (top to bottom) for $\delta =0$, $\delta =0.01$, $\delta =0.1$ (left to right)

Based on the same set of excitation combinations and the same starting value (see the beginning of Sect. 5.3), we generated the reconstructions displayed in Fig. 8. As already mentioned above, they are actually worse than those obtained in the (more nonlinear) IAT problem and the use of the CEM clearly leads to boundary artefacts.

### Numerical results for EIT

**Fig. 9:**
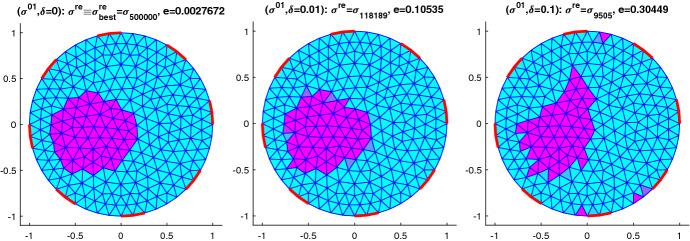
Reconstructions of $\sigma$ from eliminating-$(\varPhi ,\varPsi )$ version of cost function EIT (71), in case $I=28$, $\delta =0$, $\delta =0.01$, $\delta =0.1$ (left to right)

We close with a few plots of reconstructions for EIT. Here, since we only have boundary observations, in order to gain enough information, it is necessary to use all measurements $I=28$. Moreover, the all-at-once and eliminating-$\sigma$ versions failed to converge, so we here only provide results with the classical reduced version of EIT corresponding to the eliminating $(\varPhi ,\varPsi )$ version (71). Starting from the constant value $\sigma _{0} = \frac{1}{2} \big ( \underline{\sigma } + \overline{\sigma } \big )$ we obtain the reconstructions in Fig. 9 for noise levels of zero, one and ten per cent. Note that in view of the exponential ill-posedness of this inverse problem, the quality of reconstructions is more than reasonable for this level of data contamination. The reasons for failure of the all-at-once and eliminating-$\sigma$ versions for EIT are at least two fold: First of all, the looser bond between parameter $\sigma$ and states $(\varPhi ,\varPsi )$ in these versions would necessitate additional regularization of the state (recall that we here only regularize $\sigma$ by imposing bounds)—which could be easily added, but we did not do so here in order to keep comparability of the three different examples. Secondly, this effect is most severe in this example due to its exponential ill-posedness.

The latter, together with some regularity loss in the solution due to the CEM, is the reason for boundary artefacts showing up at the highest noise level.

More details in particular on numerical tests for EIT can be found in the PhD thesis [12].

## Conclusions and remarks

In this paper we have provided convergence results on the iterative solution methods (gradient or Newton type) for minimization based formulations of inverse problems. We apply these to the identification of a spatially varying diffusion coefficient in an elliptic PDE from different kinds of measurements, in particular corresponding to the electrical impedance tomography EIT, the impedance acoustic tomography IAT problem, and the inverse groundwater filtration problem GWF. We provide numerical results for these three test cases, thereby mainly focusing on IAT. Future work will, e.g., be concerned with investigations on the convexity conditions: How can an additive combination of functionals and constraints help to satisfy them, e.g., for EIT or IAT?

Also a comparison of the Newton type method analyzed in Sect. 3 with the gradient type method from Sect. 2 should be carried out. Clearly the Newton type method is more demanding both from an implementation and from a computational cost (per step) point of view. However, this might still pay off in view of the fact that it can be expected to reach the desired error tolerance already after a much smaller number of steps.

## Data Availability

Data sharing not applicable to this article as no datasets were generated or analysed during the current study.

## Appendix

### Lemma 2

([1, Lemma A.3]; Opial, discrete) *Let* *S* *be a non empty subset of a Hilbert space* *X*, *and* $(x_k)_{k\in \mathbb {N}}$ *a sequence of elements of* *X*. *Assume that*

(i) *for every* $z \in S$, $\lim _{k\rightarrow \infty }\Vert x_k - z\Vert$ *exists*;
(ii) *every weak sequential limit point of* $(x_k)_{k\in \mathbb {N}}$, *as* $k\rightarrow \infty$, *belongs to* *S*.

*Then* $x_k$ *converges weakly as* $k\rightarrow \infty$ *to a point in* *S*.

### Lemma 3

([1, Lemma A.2]; Opial, continuous) *Let* *S* *be a non empty subset of a Hilbert space* *X*, *and* $x:[0,\infty )\rightarrow X$ *a map. Assume that*

(i) *for every* $z \in S$, $\lim _{t\rightarrow \infty }\Vert x(t) - z\Vert$ *exists*;
(ii) *every weak sequential limit point of* *x*(*t*), *as* $t\rightarrow \infty$, *belongs to* *S*.

*Then* *x*(*t*) *converges weakly as* $t\rightarrow \infty$ *to a point in* *S*.
